# Spectral Properties of Stochastic Processes Possessing Finite Propagation Velocity

**DOI:** 10.3390/e24020201

**Published:** 2022-01-28

**Authors:** Massimiliano Giona, Andrea Cairoli, Davide Cocco, Rainer Klages

**Affiliations:** 1 Dipartimento Ingegneria Chimica Materiali Ambiente, La Sapienza Università di Roma, Via Eudossiana 18, 00184 Roma, Italy; massimiliano.giona@uniroma1.it; 2 The Francis Crick Institute, 1 Midland Road, London NW1 1AT, UK; andrea.cairoli@crick.ac.uk; 3 Dipartimento SBAI, La Sapienza Università di Roma, Via Antonio Scarpa 16, 00161 Roma, Italy; davide.cocco@uniroma1.it; 4 School of Mathematical Sciences, Queen Mary University of London, Mile End Road, London E1 4NS, UK

**Keywords:** stochastic processes, spectral properties, finite propagation velocity, Lévy walks

## Abstract

This article investigates the spectral structure of the evolution operators associated with the statistical description of stochastic processes possessing finite propagation velocity. Generalized Poisson–Kac processes and Lévy walks are explicitly considered as paradigmatic examples of regular and anomalous dynamics. A generic spectral feature of these processes is the lower boundedness of the real part of the eigenvalue spectrum that corresponds to an upper limit of the spectral dispersion curve, physically expressing the relaxation rate of a disturbance as a function of the wave vector. We also analyze Generalized Poisson–Kac processes possessing a continuum of stochastic states parametrized with respect to the velocity. In this case, there is a critical value for the wave vector, above which the point spectrum ceases to exist, and the relaxation dynamics becomes controlled by the essential part of the spectrum. This model can be extended to the quantum case, and in fact, it represents a simple and clear example of a sub-quantum dynamics with hidden variables.

## 1. Introduction

The investigation of micro- and nanoscale physics [1] as well as the extension of statistical physical concepts to new phenomelogies involving active matter and living beings [2,3] have stimulated the development of more refined descriptions of stochastic processes, accounting for background and thermal or quantum fluctuations, aimed at deriving their statistical features and long-term, in some cases anomalous [4], scaling properties [5,6]. Experimental results on the motion of bacteria, amoebae, insects, and other classes of living beings indicate that the classical paradigm of Wiener fluctuations (the mathematical Brownian motion) does not always apply to the erratic kinematics of these entities [7].

Turning our attention to a completely different branch of physics, namely field theory, the physical constraints on the texture of the space–time fabric dictate the requirement for the finite propagation velocity as an essential condition in relativistic consistent models of fluctuations [8,9]. What is remarkable in this context is that the Lorentz covariance applies to the opposite ends of the length scale spectrum, i.e., at very short (quantum fluctuations at the particle level) [10] and very large length scales (cosmological models) [11].

But even for processes in the everyday human experience, such as those related to mass, momentum, and heat transport that represent the natural realm of investigation of thermodynamic and transport theories, the quest to overcome the classical paradoxes resulting from the use of stochastic equations with infinite propagation speed—unavoidable within the assumption of constitutive equations of the Fickian type (i.e., where the flux of the thermodynamic entities is proportional and opposite to their concentration gradients, such as in the Fick, Fourier, and Newton constitutive equations for mass, heat, and momentum “diffusive” fluxes)—is not only dictated by aesthetical or epistemological arguments supporting the internal coherence of these macroscopic and statistical theories [12], but stems from the resolution of physical problems such as those of solute transport through polymeric materials or heat transport in nanodevices [13,14].

A common denominator in all these issues can be found in the understanding of the emergent properties characterizing stochastic models that possess a finite propagation velocity and of their distinctive features, either as regards the local regularity of their trajectories or their collective statistical behavior. In this article, we focus on the latter issue. Specifically, we study the spectral structure of the evolution operators that propagate the probability densities of these models. Henceforth, we refer to these operators as Statistical Evolution Operators, or SEO. This is because the constraint of bounded velocity enforces specific and characteristic spectral features, distinguishing them from their classical and widely used counterparts, namely the Wiener and Wiener-driven fluctuations. We thoroughly investigate the fluctuation spectra of Generalized Poisson-Kac processes (GPK) [15,16,17,18] and Lévy Walks (LW) [19,20,21,22], as these processes represent two of the main classes of stochastic models characterized by bounded propagation velocity.

GPK processes originate from the need to define a sufficiently wide class of stochastic models that are able to interpret the equations of extended thermodynamics for physical processes far from equilibrium at the mesoscopic level [12,23]. This represents an extension of the original model proposed by Kac [15] of a stochastic process driven by the parity of a Poisson counting process, which has been the subject of intense investigation in the past as a prototype of a non-Markovian stochastic motion driven by bounded, dichotomous, and colored noise [24,25,26,27,28]. We choose these two classes of processes because they are subjected to a common and complete statistical description in terms of the partial probability densities parametrized with respect to the internal variables of the process (for GPK, the velocity direction; for LWs, the transition age). This was shown especially for LWs by the recent work of Fedotov [29] (see also [30,31,32,33]), and by the unifying theory of Extended Poisson–Kac processes developed by Giona and collaborators [34]. Our main result is that the real part of the eigenvalues of the associated SEO of these processes is bounded from below. Consequently, any perturbation of an arbitrary wavelength cannot relax faster than an exponential function $e^{-\mu_{l}t}$, where $\mu_{l}$ is the lower bound.

This article is organized as follows. In Section 2 and Section 3, we discuss one-velocity models. These are processes characterized by a single velocity in norm, where only the direction of motion may change. These processes may seem, at a first glance, to be a simplistic physical model. However, in reality, they represent an important physical situation related to photon propagation in material media. In fact in this scenario, the transition in the velocity direction of the photon is solely dictated by scattering events [35].

In Section 4, we extend the analysis to processes characterized by a continuous structure of stochastic states. While the main qualitative result obtained in Section 2 and Section 3 is also confirmed in this case, novel and qualitatively interesting features arise, such as the incompleteness of the spectrum, and the fact that the eigenvalue spectrum can be defined solely for particular wave vectors (in the case discussed, these belong to an interval). The consequence of the latter property is that the relaxation decay in density dynamics is controlled not only by the point spectrum but also by the essential component of the spectrum. For a mathematical definition of the point and the essential spectra, the reader is referred to the classical monograph by Kato [36]. Throughout this article, we adopt an “operational” interpretation of the essential spectrum as the spectral complement to the point spectrum: an eigenvalue of the linear operator *A* defined in a functional space *F* (say the space of square summable functions) belongs to the essential spectrum if the corresponding eigenfunction does not belong to the functional space *F*.

Given the strong analogy between Poisson–Kac processes and quantum mechanics as provided by the seminal paper by Gaveau et al. [37], we explore an extension of the Gaveau model based on the functional structure of the model developed in Section 4, which possesses a continuum of velocity states, focusing on its spectral properties. This provides an interesting and exactly solvable example of a prototypical sub-quantum theory, in line with David Bohm [38,39], whose theory’s emergent properties coincide with the classical Schrödinger equation.

## 2. One-Velocity GPK Processes

**Figure 1:**
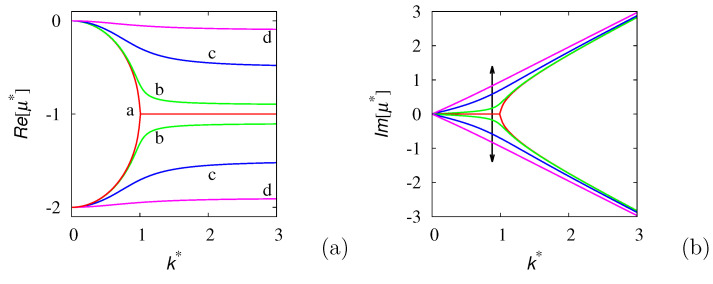
Real (**a**) and imaginary (**b**) parts of the eigenvalue spectrum $\mu^{*}$ vs. $k^{*}$ of the biased 1D GPK model at different values of the parameter *r*. Panel (**a**): line (a) refers to r=0, line (b) to r=0.1, line (c) to r=0.5, line (d) to r=0.9. Panel (**b**): the arrow indicates increasing values of *r*, the same as in panel (**a**).

Consider the simplest GPK process on the real line possessing two states s={1,2}, a uniform transition rate $\lambda_{0}$ and a transition probability matrix A given by the following equation:

(1) $$A=\frac{1}{2}(\begin{array}{ccc} 1+r & \; & 1+r\\ 1-r & \; & 1-r \end{array})\;,$$

where r∈[0,1). The velocities in the two states are equal in absolute value and opposite in sign. We denote them as $b\left(1\right)=b\left(s=1\right)=b_{0}$, $b\left(2\right)=b\left(s=2\right)=-b_{0}$. This is therefore a one-velocity model. The stochastic model can be expressed as follows:

(2) $$dx\left(t\right)=b(\chi_{2}\left(t;\lambda_{0},A\right))\;dt\;,$$

where $\chi_{2}\left(t;\lambda_{0},A\right)$ is a two-state finite Poisson process attaining the values {1,2}, i.e., a Markov process characterized by the transition rate $\lambda_{0}$ and by the transition probability matrix A. The parameter *r* in Equation (1) determines a bias in the stochastic motion. The occurrence of two stochastic states implies that the statistical description of the process involves the partial probability densities $p\left(x,t\right)=(p_{1}\left(x,t\right),p_{2}\left(x,t\right))$, and the SEO is given by ∂p(x,t)/∂t=L[p(x,t)], where the infinitesimal generator L is [18]:

(3) $$L=\left(\begin{array}{ccc} -b_{0}\frac{\partial }{\partial x}-\tilde{\lambda }\;\left(1-r\right)\; & \; & \tilde{\lambda }\;\left(1+r\right)\\ \; & \; & \\ \tilde{\lambda }\;\left(1-r\right) & \; & b_{0}\frac{\partial }{\partial x}-\tilde{\lambda }\;\left(1+r\right) \end{array}\right)\;,$$

while $\tilde{\lambda }=\lambda_{0}/2$. Consider the associated eigenvalue problem L[ψ(x)]=μψ(x). From the structure of L defined by Equation (3), the eigenfunctions are the imaginary exponentials $\psi \left(x\right)=e^{ik\;x}\left(\psi_{1}^{0},\psi_{2}^{0}\right)$. Introducing the dimensionless quantities $\mu^{*}=\mu /\tilde{\lambda }$, $k^{*}=k\;b_{0}/\tilde{\lambda }$, the eigenvalues of L can be expressed by the following relation:

(4) $$\mu^{*}=-1+{\left[1-{\left(k^{*}\right)}^{2}-i\;2\;k^{*}\;r\right]}^{1/2}\;,$$

where the square root should be interpreted in its multi-valued meaning, and $i=\sqrt{-1}$. The eigenvalue structure described by Equation (4) is depicted in Figure 1. More specifically: (i) the real part of the eigenvalues is lower bounded, i.e., Re[μ*]≥−2 (and of course, Re[μ*]≤0). For finite $\lambda_{0}$, this implies that the dynamics of any perturbation of the probability density δp(x,t), is lower bounded as $\left||\delta p\left(x,t\right)|\right|\geq Ce^{-2\;t^{*}}$, where ||·|| is any suitable norm (for instance, the $L^{2}$-norm) and $t^{*}=t\tilde{\lambda }$. For this process, therefore, $\mu_{l}=\lambda_{0}$. (ii) For any wave vector *k*, two spectral branches exist. While in the symmetric case, r=0, the real parts of the spectral branches collapse into a single one for $k^{*}>1$, the effect of a bias, r>0, is to keep the two branches separate for any $k^{*}$. This phenomenon can be understood easily in the limit $k^{*}\to \infty$. Assuming $k^{*}\gg 1$ in Equation (4), we can write $\mu^{*}≃-1+{\left[-{\left(k^{*}\right)}^{2}-i\;2\;k^{*}\;r\right]}^{1/2}$. However, ${\left(k^{*}\right)}^{2}+2\;i\;k^{*}\;r={\left(k^{*}+i\;r\right)}^{2}+r^{2}≃{\left(k^{*}+i\;r\right)}^{2}$, and consequently, $\mu^{*}=-1\pm i\left(k^{*}+i\;r\right)$. This implies that the high wave vector region of the spectrum is well-approximated by the two eigenvalue branches $\mu^{*}=-\left(1+r\right)+i\;k^{*}$ and $\mu^{*}=-\left(1-r\right)-i\;k^{*}$, thus explaining the qualitative features depicted in Figure 1.

**Figure 2:**
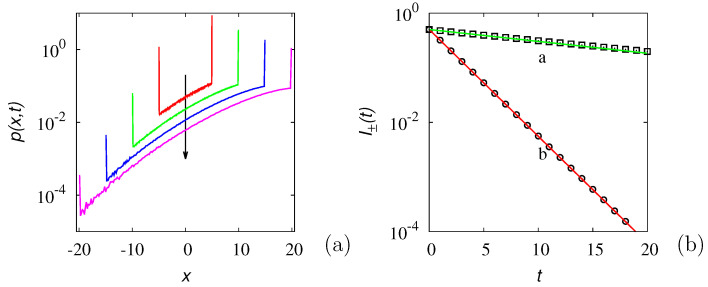
(**a**) Overall probability density p(x,t) vs. *x* of the biased 1D GPK model, with $b_{0}=1$, D=1, at r=0.8, starting from symmetric, impulsive initial conditions. The arrow indicates increasing time instants t=5,10,15,20. (**b**) Decay with time of the two impulsive components $I_{\pm }\left(t\right)$ of the overall Green function. Symbols are the results of stochastic simulations, lining the predictions e−Re[μ1,2∞]t. Line (a) and (□): $I_{+}\left(t\right)$; line (b) and (∘): $I_{-}\left(t\right)$.

The latter result finds a direct interpretation in terms of the salient properties of the Green functions for this class of random motion. Consider the dynamics of an ensemble of particles moving according to Equation (2) and starting from the origin. Assume symmetric initial conditions, i.e., $p_{1}\left(x,0\right)=p_{2}\left(x,0\right)=\delta \left(x\right)/2$. Figure 2a depicts the evolution of the particle ensemble, expressed in terms of the overall probability density function, $p\left(x,t\right)=p_{1}\left(x,t\right)+p_{2}\left(x,t\right)$, obtained from stochastic simulations of $N_{p}=5\times 10^{7}$ particles with $b_{0}=1$, $\lambda_{0}=1/2$, so that $D=b_{0}^{2}/2\lambda_{0}=1$. A characteristic feature of this class of finite-velocity stochastic motions is that the diagonal entries of the matrix-valued Green function are characterized by the superposition of a continuous, compactly supported component and of an impulsive contribution. Expressed in terms of the overall probability density p(x,t) starting from an impulsive initial condition, this implies that $p\left(x,t\right)=p^{c}\left(x,t\right)+p^{\delta }\left(x,t\right)$, where $p^{c}\left(x,t\right)$ is the continuous term and $p^{\delta }\left(x,t\right)$ is the impulsive one, namely $p^{\delta }\left(x,t\right)=I_{+}\left(t\right)\;\delta \left(x-b_{0}\;t\right)+I_{-}\left(t\right)\delta \left(x+b_{0}\;t\right)$. The functions $I_{\pm }\left(t\right)$ progressively fade away as *t* increases [40,41,42]. The previous discussion suggests that the decay of the impulsive branches is controlled by the real part of the two asymptotic eigenvalues Re[μ1∞]=−λ˜(1+r) for $I_{-}\left(t\right)$ and Re[μ2∞]=−λ˜(1−r) for $I_{+}\left(t\right)$. This phenomenon is depicted in Figure 2b.

Next, we consider a two-dimensional GPK model possessing *N* stochastic states. This process can be characterized in terms of *N* partial probability densities $p\left(x,t\right)={\left(p_{\alpha }\left(x,t\right)\right)}_{\alpha =1}^{N}$, satisfying the following general evolution equation [18]:

(5) $$\frac{\partial p_{\alpha }}{\partial t}=-b_{\alpha }\cdot \nabla p_{\alpha }-\lambda_{\alpha }\;p_{\alpha }+\sum_{\beta =1}^{N}A_{\alpha ,\beta }\;\lambda_{\beta }\;,\;p_{\beta }$$

with transition rates among states $\lambda_{\alpha }>0$, a probability transition matrix $A_{\alpha ,\beta }\geq 0$, where $\sum_{\alpha =1}^{N}A_{\alpha ,\beta }=1$ for α,β=1,⋯,N, and velocity vectors $b_{\alpha }$. Moreover, in this case, we are interested in the spectral properties of the associated SEO (now vector-valued) acting on the partial densities defined by Equation (5). The eigenfunctions are planar waves, $\psi_{\alpha }\left(x\right)=e^{i\;k\cdot x}\;\psi_{\alpha }^{0}$, for α=1,⋯,N. As regards the internal structure of the GPK model, we assume that all the transition rates are equal $\lambda_{\alpha }=\lambda_{0}$, the transition probability matrix is uniform $A_{\alpha ,\beta }=1/N$, and the characteristic velocity vectors are $b_{\alpha }=b_{0}\;\beta_{\alpha }$, with $\beta_{\alpha }=\left(\cos\varphi_{\alpha },\sin\varphi_{\alpha }\right)$ and $\varphi_{\alpha }=2\;\pi \left(\alpha -1\right)/N$ for α=1,⋯,N. Introducing the non-dimensional quantities, $k^{*}=k\;b_{0}/\lambda_{0}$, $\mu^{*}=\mu /\lambda_{0}$, we can write the eigenvalue equation as follows:

(6) $$\sum_{\beta =1}^{N}\left[A_{\alpha ,\beta }-\left(ik^{*}\cdot \beta_{\alpha }+1\right)\;\delta_{\alpha ,\beta }\right]\;\psi_{\beta }^{0}=\mu^{*}\;\psi_{\alpha }^{0}\;,$$

where $\delta_{\alpha ,\beta }$ are the Kronecker symbols.

**Figure 3:**
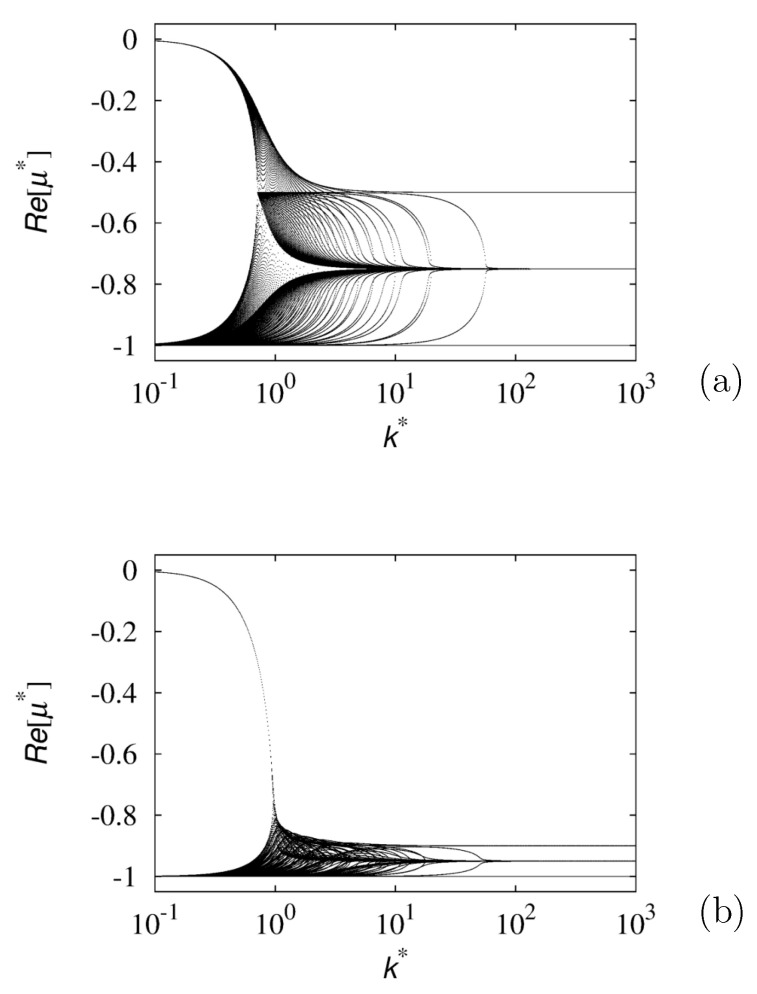
Real part of the spectrum vs. the wave vector $k^{*}$ for the 2D GPK model discussed in the text with *N* stochastic states. (**a**): N=4; (**b**): N=20.

Figure 3 shows the real part of the eigenvalue spectrum of these models for two different values of *N* as a function of the norm $k^{*}=\left|k^{*}\right|$ of the wave vector. The real part of the eigenvalue spectrum is lower bounded, specifically Re[μ*]≥−1. For this 2D GPK process, therefore, we obtain the same lower bound of the 1D biased model, $\mu_{l}=\lambda_{0}$. The comparison of the data shown in the two panels suggests the existence of a well-defined spectral limit for N→∞, i.e., in the case where the velocities are uniformly distributed on the unit circumference. This limiting case, which corresponds to the Markov motions analyzed by Kolesnik [17], suggests that for a continuum of stochastic states, the spectral properties of the resulting SEO give rise to a much simpler structure for the spectral branches.

## 3. Lévy Walks

Owing to the analysis developed by Fedotov [29], subsequently elaborated in [30,31,32,33] and extended in a unitary theory of stochastic processes possessing bounded velocity in [34], a complete statistical description of an LW involves a system of partial probability densities, as for GPK processes. The only difference is that in the case of LW, we also need to parametrize the partial densities with respect to an internal parameter, the transition age. This is due to the more complex nature of the density function for the transition times that is no longer exponential (as for Poisson–Kac processes). We consider a one-dimensional LW switching between two states, corresponding to the directions of motion, keeping constant the absolute value of the velocity $b_{0}$. The statistical properties of such an LW are fully described by the partial probability densities $p_{\pm }\left(x,t;\tau \right)$. Setting the following equation:

(7) $$p\left(x,t;\tau \right)=\left(\begin{array}{c} p_{+}\left(x,t;\tau \right)\\ p_{-}\left(x,t;\tau \right) \end{array}\right)\;,$$

the vector-valued density p(x,t;τ) satisfies the evolution equation [29]

(8) $$\frac{\partial p}{\partial t}=M_{\tau }\left[p\right]+A_{x}\left[p\right]=L_{\tau ,x}\left[p\right]\;,$$

where $M_{\tau }$ represents the evolution operator for the transition age,

(9) $$M_{\tau }=\left(\begin{array}{cc} -\frac{\partial }{\partial \tau }-\lambda \left(\tau \right) & 0\\ 0 & -\frac{\partial }{\partial \tau }-\lambda \left(\tau \right) \end{array}\right)=-I_{2}\left(\frac{\partial }{\partial \tau }+\lambda \left(\tau \right)\right)\;,$$

$I_{2}$ is the 2×2 identity matrix, and $A_{x}$ represents the advection operator:

(10) $$A_{x}=\left(\begin{array}{cc} -b_{0}\;\frac{\partial }{\partial x} & 0\\ 0 & b_{0}\;\frac{\partial }{\partial x} \end{array}\right)=-\sigma_{z}\;b_{0}\;\frac{\partial }{\partial x}\;,$$

with $\sigma_{z}=\left(\begin{array}{cc} 1 & 0\\ 0 & -1 \end{array}\right)$, a Pauli matrix. Both operators act on the two partial probability density waves $p_{\pm }$. Assuming that particles invert their direction of motion at each transition, Equation (8) is equipped with the following boundary condition:

(11) $$p\left(x,t,0\right)=\sigma_{x}\;\int_{0}^{\infty }\lambda \left(\tau \right)\;p\left(x,t,\tau \right)\;d\tau \;,$$

where $\sigma_{x}$ is the Pauli matrix $\sigma_{x}=\left(\begin{array}{cc} 0 & 1\\ 1 & 0 \end{array}\right)$. We now consider the spectral structure underlying the SEO, that is, we solve the eigenvalue problem:

(12) $$L_{\tau ,x}\left[\psi \left(x,\tau \right)\right]=\mu \;\psi \left(x,\tau \right)\;,\;\psi \left(x,\tau \right)=\left(\begin{array}{c} \psi_{+}\left(x,\tau \right)\\ \psi_{-}\left(x,\tau \right) \end{array}\right)\;.$$

The eigenfunction ψ(x,τ) can be expressed as planar waves with respect to the spatial coordinate, i.e., $\psi \left(x,\tau \right)=e^{i\;k\;x}\phi \left(\tau \right)$, while the dependence on the transition time τ is accounted for by the vector-valued function $\phi \left(\tau \right)=(\phi_{+}\left(\tau \right),\phi_{-}\left(\tau \right))$. Its two components satisfy the following equations:

(13) $$\frac{d\phi_{\pm }\left(\tau \right)}{d\tau }=-\left(\mu \pm i\;k\;b_{0}+\lambda \left(\tau \right)\right)\;\phi_{\pm }\left(\tau \right)\;,$$

and thus,

(14) $$\phi_{\pm }\left(\tau \right)=A_{\pm }\;\exp\left[-\mu \;\tau \mp i\;k\;b_{0}\;\;\tau -\int_{0}^{\tau }\lambda \left(\theta \right)\;d\theta \right]\;,$$

where $A_{\pm }$ are two complex-valued constants.

Imposing the boundary conditions (11) on the function ϕ, we find that the two constants $A_{\pm }$ satisfy the following relation:

(15) $$A_{\pm }=A_{\mp }\;\int_{0}^{\infty }T\left(\tau \right)e^{-(\mu \pm i\;k\;b_{0})\;\tau }\;d\tau \;,$$

where $T\left(\tau \right)=\lambda \left(\tau \right)\;\exp\left[-\int_{0}^{\tau }\lambda \left(\theta \right)\;d\theta \right]$ is the probability density function for the transition times. Eliminating $A_{\pm }$ from the two in Equation (15), we obtain the characteristic equation for the eigenvalues of $L_{\tau ,x}$, given as follows:

(16) $$\left[\int_{0}^{\infty }T\left(\tau \right)e^{-(\mu +i\;k\;b_{0})\;\tau }\;d\tau \right]\left[\int_{0}^{\infty }T\left(\tau^{\prime }\right)e^{-\left(\mu -i\;k\;b_{0}\right)\;\tau^{\prime }}\;d\tau^{\prime }\right]=1\;.$$

For k=0, one recovers the spectral properties of the “renewal mechanism” of the LW. In this case, Equation (16) reduces to the following:

(17) $${\left[\int_{0}^{\infty }T\left(\tau \right)e^{-\mu \;\tau }\;d\tau \right]}^{2}=1\;.$$

Equation (17) can be interpreted as follows: the spectral structure of the renewal equation of an LW corresponds to the zeroes of the equations $\hat{T}\left(\mu \right)=\pm 1$, where $\hat{T}\left(\mu \right)$ represents the Laplace transform (of argument μ) of the transition-time probability density T(τ). For real μ, only the positive determination of the latter equation makes sense, and it can be clearly noted that there exists a unique real solution for $\hat{T}\left(\mu \right)=1$, namely μ=0, that corresponds to the Frobenius (conservation) eigenvalue of the process.

In the rest of this section, we consider three typical LW models and solve for their spectral properties.

### 3.1. Gamma-Distributed Transition Times

For this model, the transition-time density is expressed by the following equation:

(18) $$T\left(\tau \right)=\frac{\beta^{\alpha }}{\Gamma (\alpha )}\;\tau^{\alpha -1}\;e^{-\beta \;\tau }\;,$$

with α∈(0,∞), β>0. Since

(19) $$\hat{T}\left(s\right)=\frac{\beta^{\alpha }}{{\left(s+\beta \right)}^{\alpha }}\;,$$

it follows that Equation (16) reduces to

(20) $${\left[{\left(\mu +\beta \right)}^{2}+k^{2}\;b_{0}^{2}\right]}^{\alpha }=\beta^{2\alpha }\;,$$

or equivalently,

(21) $${\left(\mu +\beta \right)}^{2}+k^{2}\;b_{0}^{2}=\beta^{2}e^{i\;\varphi_{\alpha ,h}}\;,$$

where $\varphi_{\alpha ,h}=2\;\pi \;h/\alpha$, and *h* is an integer, h=0,1,⋯. Two cases may occur: (i) if α=P/Q is rational, with P,Q both being integers, there are *Q* distinct values for $e^{i\;\varphi_{\alpha ,h}}$ that correspond to h=0,1,⋯,Q; (ii) if α is irrational, there is a countable number of distinct values for $e^{i\;\varphi_{\alpha ,h}}$, each corresponding to a different spectral branch.

The spectral properties of the renewal mechanism (corresponding to k=0) follow from Equation (21):

(22) $$\mu_{h}=-\beta \pm \beta e^{i\;\varphi_{\alpha ,h}/2}\;,$$

while the eigenvalues are generally complex-valued. In particular, if α is irrational, $(\mu_{h}+\beta )/\beta$, h=0,1,⋯, densely fill the unit circumference.

Next, we consider the case k≠0. Solving the quadratic Equation (21), one obtains the following:

(23) $$\mu_{h}=-\beta \pm {\left[\beta^{2}e^{i\;\varphi_{\alpha ,h}}-k^{2}\;b_{0}^{2}\right]}^{1/2}=-\beta \pm \sqrt{z_{h}}\;,$$

where $z_{h}=\left[\beta^{2}\cos\left(\varphi_{\alpha ,h}\right)-k^{2}\;b_{0}^{2}\right]+i\;\beta^{2}\;\sin\left(\varphi_{\alpha ,h}\right)=\rho_{h}\;e^{i\;\omega_{h}}$. Since the two determinations of $\sqrt{z_{h}}$ are $\pm \rho_{h}^{1/2}e^{i\;\omega_{h}/2}$, it follows that:

(24) $$\mu_{h}=-\beta \pm \rho_{h}^{1/2}\;\left[\cos\left(\frac{\omega_{h}}{2}\right)+i\;\sin\left(\frac{\omega_{h}}{2}\right)\right]\;.$$

It can be clearly shown that the real part of the spectrum is lower bounded. Moreover, the explicit calculation of the eigenvalues indicates that:

(25) −2β≤Re[μh]≤0.

**Figure 4:**
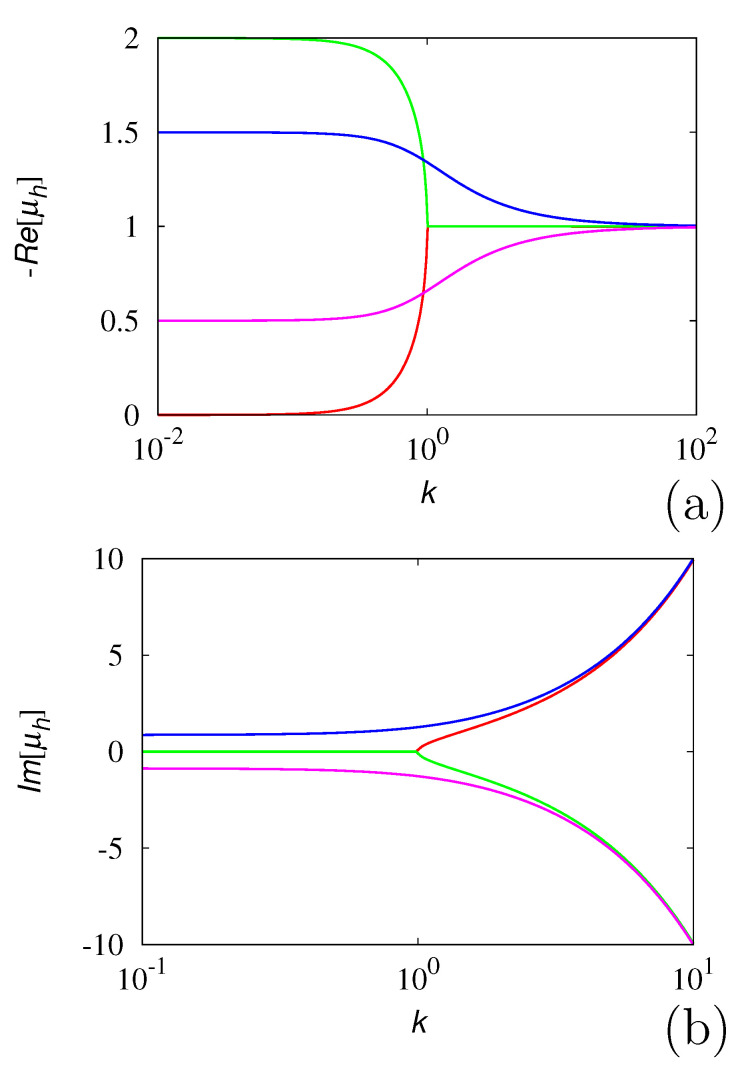
Spectral structure of the Gamma-distributed LW at α=3, $\beta =b_{0}=1$ a.u. (**a**): −Re[μh] vs. *k*; (**b**): $Im[\mu_{h}]$ vs. *k*.

**Figure 5:**
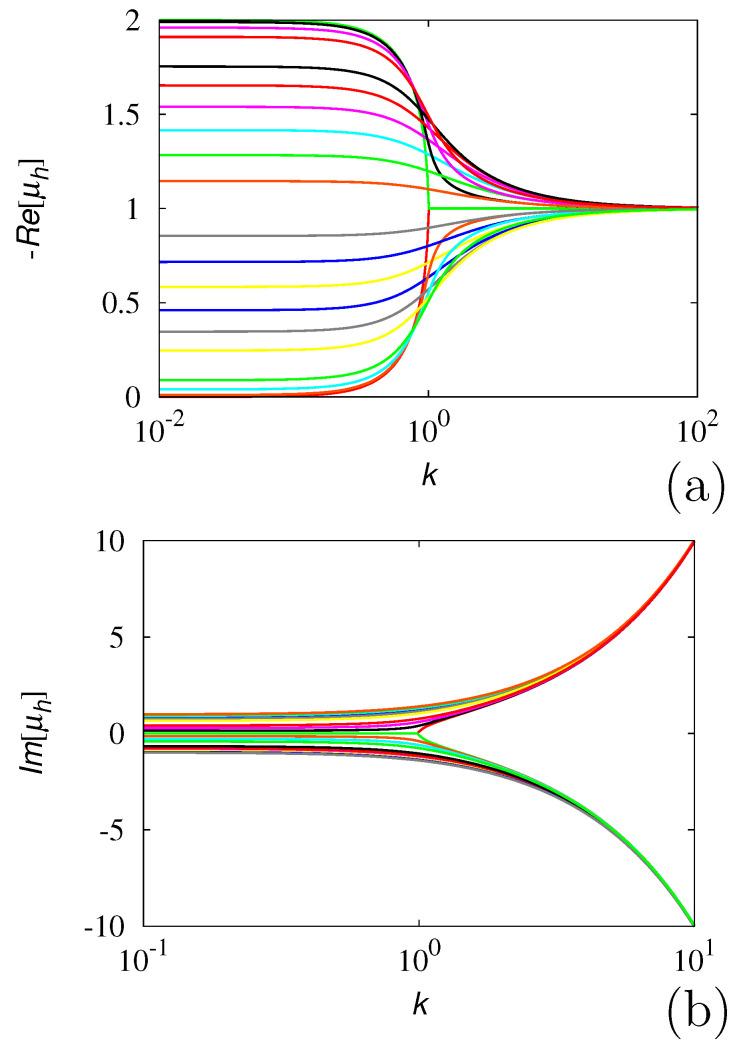
Spectral structure of the Gamma-distributed LW at α=π, $\beta =b_{0}=1$ a.u. (**a**): −Re[μh] vs. *k*; (**b**): Im[μh] vs. *k*. Solely the first 20 spectral branches are depicted.

In order to give some examples, Figure 4 and Figure 5 depict the real and imaginary part of the spectrum in two typical cases: α=3 (integer) and α=π (irrational). The other parameters are set to $\beta =b_{0}=1$. In the irrational case, solely the the spectral branches corresponding to h=0,⋯,19, are depicted.

### 3.2. Superdiffusive Lévy Walk

For LWs defined by a transition-time probability density possessing a long-term power-law scaling, such as those associated with the distribution function $F_{\tau }\left(\tau \right)=\int_{0}^{\tau }T\left(\tau^{\prime }\right)\;d\tau^{\prime }=1-E_{\nu }\left(-{\left(\tau /\tau_{0}\right)}^{\nu }\right)$, where $E_{\nu }\left(z\right)=\sum_{k=0}^{\infty }z^{k}/\Gamma \left(\nu \;k+1\right)$ is the Mittag–Leffler function, 0<ν<1, $\tau_{0}>0$, and Γ(z) the Gamma function [43,44], the Laplace transform of T(τ) is thus given by the following equation:

(26) $$\hat{T}\left(s\right)=\frac{1}{1+{\left(\tau_{0}\;s\right)}^{\nu }}\;,$$

and Equation (16) provides:

(27) $$\left[1+\tau_{0}^{\nu }\;{\left(\mu +i\;k\;b_{0}\right)}^{\nu }\right]\;\left[1+\tau_{0}^{\nu }\;{\left(\mu -i\;k\;b_{0}\right)}^{\nu }\right]=1\;.$$

To solve this equation, we set the following:

(28) μ+ikb0=Re[μ]+iIm[μ]±kb0=ρeiϕ±,

where ρ≥Re[μ]. Equation (27) can be thus rewritten as follows:

(29) $$1+\tau_{0}^{\nu }\;\rho^{\nu }\;\left(e^{i\;\nu \;\phi_{+}}+e^{i\;\nu \;\phi_{-}}\right)+\tau_{0}^{2\;\nu }\rho^{2\;\nu }\;e^{i\;\nu \;(\phi_{+}+\phi_{-})}=1\;,$$

which can be further reduced to the equation below:

(30) $$\tau_{0}^{\nu }\;\rho^{\nu }+\left(e^{-i\;\nu \;\phi_{+}}+e^{-i\nu \phi_{-}}\right)=0\;.$$

This implies the following relation:

(31) |Re[μ]|≤ρ≤21/ντ0,

which proves the boundedness of the real part of the spectrum.

### 3.3. Power-Law Distributed Transition Times

We consider as a third example the classical case of an LW (8)–(11) defined by the the transition-time probability density $T\left(\tau \right)=\xi /{\left(1+\tau \right)}^{\xi +1}$, where ξ>0. Its Laplace transform is given by the following equation:

(32) $$\hat{T}\left(\mu \right)=\xi \;\int_{0}^{\infty }\frac{e^{-\mu \;\tau }}{{\left(1+\tau \right)}^{\xi +1}}\;d\tau =\xi \;e^{\mu }\;{Ei}_{\xi +1}\left(\mu \right)\;,$$

where ${\mathrm{Ei}}_{\xi +1}\left(z\right)$ is the Exponential Integral function of order ξ+1,

(33) $${\mathrm{Ei}}_{\nu }\left(z\right)=\int_{1}^{\infty }\frac{e^{-zy}}{y^{\nu }}\;dy\;,$$

In this case, it is not possible to derive the structure of the eigenvalue branches in closed form. Nevertheless, we can still obtain spectral bounds using asymptotic analysis.

Let us prove *ad absurdum* that there exists a lower bound for the real part of the eigenvalue spectrum. Let us suppose the opposite, namely that there are eigenvalues with arbitrarily large real parts, which implies arbitrarily large values for |μ|. In this case, we can use Equation (32), and the asymptotic expansion for the Exponential Integral function ${\mathrm{Ei}}_{\nu }\left(z\right)$ for complex *z* [45]:

(34) $${\mathrm{Ei}}_{\nu }\left(z\right)=\frac{e^{-z}}{z}\left[1-\frac{\nu }{z}+O\left(\frac{1}{z^{2}}\right)\right]\;,$$

which implies the following:

(35) $$\hat{T}\left(\mu \pm i\;k\;b_{0}\right)=\frac{\xi }{\mu \pm i\;k\;b_{0}}+{\left.O\left(\frac{1}{s^{3}}\right)\right|}_{s=\mu \pm i\;k\;b_{0}}\;.$$

For large Re[μ], one obtains the following from Equation (16):

(36) ξ2(μ+ikb0)(μ−ikb0)=1+O1Re[μ]4.

By hypothesis, the real part of the eigenvalues can attain arbitrarily large values so that the higher-order terms in Equation (36) can be neglected, reducing it to the equation below:

(37) $$\mu^{2}+k^{2}\;b_{0}^{2}-\xi^{2}=0\;.$$

The solutions of this equation are as follows:

(38) $$\mu =\left\{\begin{array}{ccc} \pm \sqrt{\xi^{2}-k^{2}\;b_{0}^{2}} & \; & |k|<\xi /b_{0}\\ \pm i\;\sqrt{k^{2}\;b_{0}^{2}-\xi^{2}} & \; & |k|>\xi /b_{0}\;. \end{array}\right.$$

It thus follows from Equation (38) that the real part of the spectrum is lower-bounded by $-\xi /b_{0}$, contradicting the original hypothesis. The proof is completed.

From the properties of the Laplace transforms, namely limRe[μ]→∞T^(μ)=0, and applying the same analysis developed above, we can show that all the one-dimensional LWs, whose spectrum fulfills Equation (16), are characterized by a lower bound $-\mu_{l}$, with $\mu_{l}>0$ for Re[μ].

## 4. GPK Process with a Continuum of States

A common feature of one-velocity models is the decomposition of the Green function into a continuous part and an impulsive part, as discussed in [40,42] and illustrated in Figure 2.

A question naturally arises, as to whether it would be possible to define a stochastic process with a finite propagation velocity, for which the corresponding matrix-valued Green function solely admits the continuous part but not an impulsive contribution, similarly to what happens to the solution of the parabolic diffusion equation. While the answer to this question is negative (for reasons that are addressed below), it is indeed possible to construct processes such that if the overall initial density is impulsive, say p(x,0)=δ(x), then for any t>0, the overall density function p(x,t) is smooth and compactly supported, i.e., it does not not possess any impulsive term. This construction is interesting when further addressing the spectral properties of these models, as the sudden annihilation of the impulsive initial conditions necessarily implies a spectral counterpart in the high-wave vector limit. Examples of these models have been discussed in [46].

Processes that possess this property implies a continuous set of stochastic states possessing velocities defined in some bounded and continuous set (in the present case, an interval) and smooth transitions among them. In this case, the velocity itself can be used to parametrize the stochastic states. A typical example is given by the stochastic dynamics:

(39) $$dx\left(t\right)=b_{0}\;\Xi \left(t;\lambda ;K\right)\;dt\;,$$

where Ξ(t;λ,K) is a Poisson field defined in [47], and attaining values in [−1,1], i.e., a stochastic process such that the densities $\hat{P}\left(\beta ,t\right)$ of the probabilities, where $\hat{P}\left(\beta ,t\right)\;d\beta =\mathrm{Prob}\left[\Xi \left(t\right)\in \left(\beta ,\beta +d\beta \right)\right]$, satisfy a Markov dynamics:

(40) $$\frac{\partial \hat{P}\left(\beta ,t\right)}{\partial t}=-\lambda \left(\beta \right)\;\hat{P}\left(\beta ,t\right)+\int_{-1}^{1}K\left(\beta ,\beta^{\prime }\right)\;\lambda \left(\beta^{\prime }\right)\;\hat{P}\left(\beta^{\prime },t\right)\;d\beta^{\prime }\;,$$

where λ(β) is a smooth positive function expressing the transition rate from the stochastic state β, and $K(\beta ,\beta^{\prime })$ a smooth stochastic kernel, with $K(\beta ,\beta^{\prime })\geq 0$, and

(41) $$\int_{-1}^{1}K\left(\beta ,\beta^{\prime }\right)d\beta =1\;,$$

for all $\beta^{\prime }\in \left[-1,1\right]$. The latter accounts for the transition probabilities from state $\beta^{\prime }$ to all the other states. Henceforth, we consider the simplest case of uniform λ(β) and $K(\beta ,\beta^{\prime })$, i.e.,:

(42) $$\lambda \left(\beta \right)=\lambda_{0}\;,\;K\left(\beta ,\beta^{\prime }\right)=\frac{1}{2}\;.$$

The statistical description of the stochastic motion defined by Equation (39) involves the family of partial probability densities p(x,t;β), continuously parametrized with respect to β∈[−1,1] and satisfying the following equation:

(43) $$\frac{\partial p(x,t;\beta )}{\partial t}=-b_{0}\;\beta \;\frac{\partial p(x,t;\beta )}{\partial x}-\lambda_{0}\;p\left(x,t;\beta \right)+\frac{\lambda_{0}}{2}\int_{-1}^{1}p\left(x,t;\beta^{\prime }\right)\;d\beta^{\prime }\;.$$

In this model, the overall probability density P(x,t) for the particle position *x* at time *t* and the associated flux J(x,t) are given by the equation below:

(44) $$P\left(x,t\right)=\int_{-1}^{1}p\left(x,t;\beta \right)\;d\beta \;,\;J\left(x,t\right)=\int_{-1}^{1}\beta \;p\left(x,t;\beta \right)\;d\beta \;.$$

Using the method outlined in [18,47,48], it follows that the Kac limit of Equation (43), i.e., the limit for the unbounded propagation velocity and the transition rate, $b_{0},\;\lambda_{0}\to \infty$, keeping fixed the nominal diffusivity $D_{\mathrm{nom}}=b_{0}^{2}/2\;\lambda_{0}$ is given by the diffusion equation for P(x,t):

(45) $$\frac{\partial P(x,t)}{\partial t}=D\;\frac{\partial^{2}P\left(x,t\right)}{\partial x^{2}}\;,$$

with a value of the effective diffusivity *D* equal to $D=2\;D_{\mathrm{nom}}/3$. Moreover, for any finite value of $b_{0}$ and $\lambda_{0}$, the long-term solutions of Equation (43) approach those of the parabolic Equation (45).

**Figure 6:**
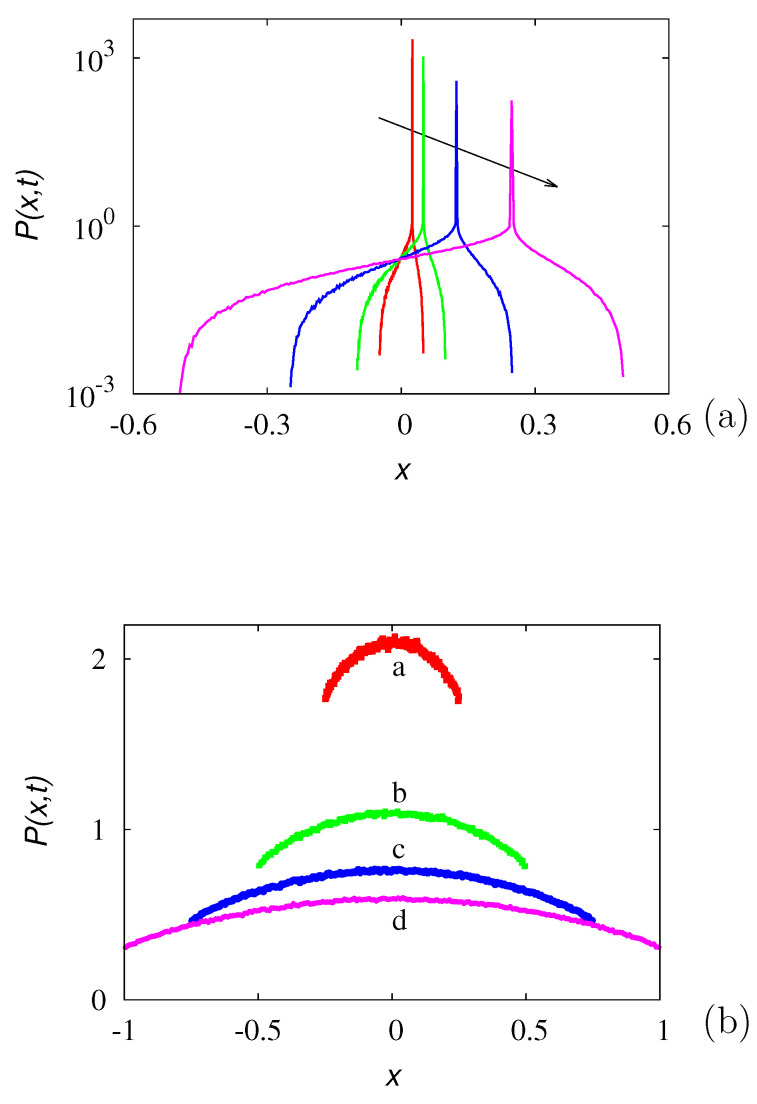
Overall probability density function P(x,t) vs. *x* associated with the process defined by Equations (39) and (42) at $b_{0}=1$, $\lambda_{0}=1/2$, with P(x,0)=δ(x), for two different initial preparations. (**a**) refers to an impulsive initial distribution ${\tilde{p}}_{0}\left(\beta \right)=\delta \left(\beta -0.5\right)$, and the arrow indicates increasing values for time t=0.05,0.1,0.25,0.5. (**b**) refers to a smooth and uniform initial preparation ${\tilde{p}}_{0}\left(\beta \right)=1/2$. Points (a) correspond to t=0.25, (b) to t=0.5, (c) to t=0.75, (d) to t=1.0.

Consider the solutions of Equation (43), starting from a spatially impulsive initial distribution P(x,0)=δ(x). This condition does not completely specify the initial state of the system, as the initial preparation with respect to the internal parametrization β should also be defined, i.e., the whole structure of the partial densities $p\left(x,0;\beta \right)={\tilde{p}}_{0}\left(\beta \right)\;\delta \left(x\right)$, β∈[−1:1] at t=0 should be specified. Two situations can occur: (i) if ${\tilde{p}}_{0}\left(\beta \right)=\delta \left(\beta -\beta^{*}\right)$ admits an impulsive component at any $\beta =\beta^{*}$, then P(x,t) for t>0 is characterized by an impulsive contribution centered at $x=\beta^{*}\;t$, propagating with velocity $\beta^{*}$ superimposed onto a continuous distribution that derives from the recombination mechanism among the partial waves. Conversely, (ii) if ${\tilde{p}}_{0}\left(\beta \right)$ is smooth, i.e., no impulsive initial term is present, then P(x,t) for t>0 is also a smooth function of *x* for any t>0. The phenomena outlined above are depicted in Figure 6a,b. Data have been obtained from stochastic simulations using an ensemble of $N_{p}=5\times 10^{7}$ walkers initially placed at x=0. In the case of the data depicted in panel (a), the initial distribution with respect to the internal state variable is impulsive and centered at $\beta^{*}=1/2$. Conversely, the initial condition for the data depicted in panel (b) is uniform over β, i.e., ${\tilde{p}}_{0}\left(\beta \right)=1/2$, β∈[−1,1].

This example clearly indicates that even in the presence of a continuum of internal states, any initial preparation that is impulsive with respect to the internal-state parametrization β determines an overall density P(x,t) that contains an impulsive contribution with respect to *x*, propagating at constant speed. It is interesting to analyze how this phenomenology can be interpreted in the light of the spectral properties of the associated evolution operator.

### 4.1. Spectral Properties

Consider the spectral properties of the infinitesimal generator of the density dynamics (43):

(46) $$L_{\beta }\left[\phi \left(x,\beta \right)\right]=-b_{0}\;\beta \;\frac{\partial \phi (x,\beta )}{\partial x}-\lambda_{0}\;\phi \left(x,\beta \right)+\frac{\lambda_{0}}{2}\int_{-1}^{1}\phi \left(x,\beta^{\prime }\right)\;d\beta^{\prime }\;.$$

The eigenvalue equation for $L_{\beta }$ is as follows:

(47) $$L_{\beta }\left[\psi \left(x,\beta \right)\right]=\mu \;\psi \left(x,\beta \right)\;,$$

which admits the eigenfunctions of the following form:

(48) $$\psi \left(x,\beta \right)=e^{i\;k\;x}\;\psi_{k}\left(\beta \right)\;.$$

Introducing the dimensionless quantities $\mu^{*}=\mu /\lambda_{0}$, $k^{*}=k\;b_{0}/\lambda_{0}$, the substitution of Equation (48) into Equations (46) and (47) provides the following equation:

(49) $$\psi_{k}\left(\beta \right)=\frac{1}{2\;(1+\mu^{*}+i\;k^{*}\;\beta )}\int_{-1}^{1}\psi_{k}\left(\beta^{\prime }\right)\;d\beta^{\prime }\;.$$

Consequently, integrating over β and assuming $\int_{-1}^{1}\psi_{k}\left(\beta \right)\;d\beta \neq 0$, the characteristic equation for the eigenvalues is as follows:

(50) $$\int_{-1}^{1}\frac{d\beta }{1+\mu^{*}+ik^{*}\;\beta }=2\;.$$

Setting $\mu^{*}=r+i\;\omega$ and expliciting the integral in Equation (50), one obtains the following equation:

(51) $$\arctan\left(\frac{\omega +k^{*}}{1+r}\right)-\arctan\left(\frac{\omega -k^{*}}{1+r}\right)-\frac{i}{2}\log\left[\frac{{\left(1+r\right)}^{2}+{\left(\omega +k^{*}\right)}^{2}}{{\left(1+r\right)}^{2}+{\left(\omega -k^{*}\right)}^{2}}\right]=2\;k^{*}\;.$$

Imposing the imaginary part to be null, one obtains ω=0, i.e., the eigenvalues are purely real, $\mu^{*}=r$. Enforcing this property back in Equation (51), a simpler equation for the real part *r* follows:

(52) $$k^{*}=\arctan\left(\frac{k^{*}}{1+r}\right)\;.$$

The solution of Equation (52) can be easily obtained by introducing the auxiliary variable $z=k^{*}/\left(1+r\right)$, so that Equation (52) becomes (1+r)z=arctan(z). Varying z∈[0,∞), one gets the continuous branch of eigenvalues:

(53) $$r=-1+\frac{\arctan(z)}{z}\;.$$

Given *z*, the corresponding wavenumber $k^{*}$ follows from the definition of *z*, namely $k^{*}=\left(1+r\right)\;z$. Before addressing the properties of this spectral branch, let us show that there is no other eigenvalue. To show this, we have to consider the only remaining condition:

(54) $$\int_{-1}^{1}\psi_{k}\left(\beta \right)\;d\beta =0\;.$$

**Figure 7:**
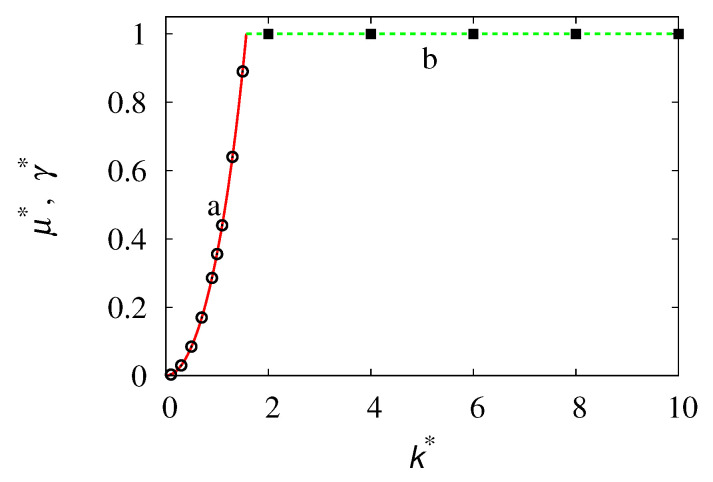
Eigenvalue branch $\mu^{*}$ vs. the wavenumber $k^{*}$ (line a). Line (b) represents the maximum relaxation exponent $\gamma^{*}=1$, associated with the essential spectrum of the evolution operator. Symbol (∘) and (*■*) are the results of the numerical simulations of Equation (57), considering the decay of the $L^{2}$ norm of the solution.

**Figure 8:**
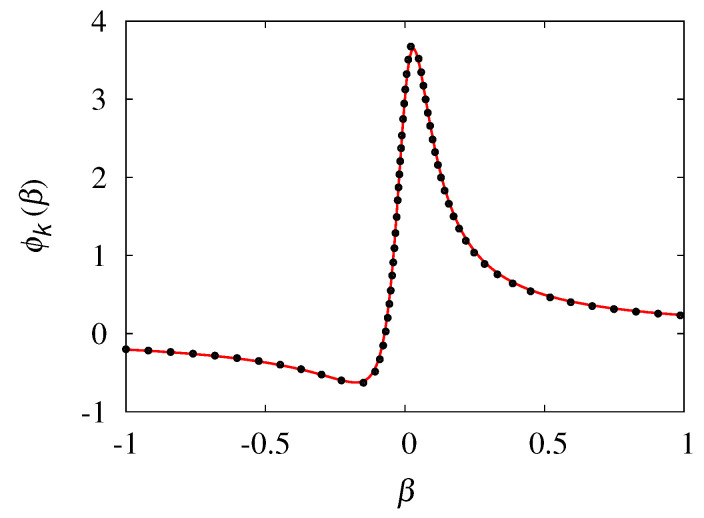
Profile of the real part of the eigenfunction $\phi_{k}\left(\beta \right)$ vs. β at $k^{*}=1.5$. The line represents the graph of Equation (56), while the symbol (•) represents the profile obtained from the relaxation of Equation (57), integrated numerically.

In this case, the eigenvalue problem reduces to $\left(\mu^{*}+1+i\;k^{*}\;\beta \right)\;\psi_{k}\left(\beta \right)=0$, which admits no solution for a $\mu^{*}$ independent of β. Consequently, the spectrum reduces to the spectral branch defined by Equations (52) and (53). This branch is defined for $|k^{*}|<k_{c}=\pi /2$, as depicted in Figure 7. In this interval of wave vectors, the associated eigenfunctions defined by Equation (49) are complex-valued and can be expressed as follows:

(55) $$\psi_{k}\left(\beta \right)=A\left[\phi_{k}\left(\beta \right)+i\phi_{k}\left(-\beta \right)\right]\;,$$

where *A*, is a complex-valued constant and $\phi_{k}\left(\beta \right)$ is a real-valued function defined as:

(56) $$\phi_{k}\left(\beta \right)=C\;\frac{\left(1+\mu^{*}\right)+k^{*}\;\beta }{{\left(1+\mu^{*}\right)}^{2}+{\left(k^{*}\;\beta \right)}^{2}}\;,$$

where the normalization constant *C* is such that $\left|\right|\phi_{k}\left(\beta \right){\left|\right|}_{L^{2}}=\int_{-1}^{1}\phi_{k}^{2}\left(\beta \right)\;d\beta =1$. Expression (55) with $\phi_{k}\left(\beta \right)$ given by Equation (56) follows from Equation (49), i.e., $\psi_{k}\left(\beta \right)=B/\left[\left(1+\mu^{*}\right)+i\;k^{*}\;\beta \right]$, setting B=1+i, modulo being an arbitrary multiplicative constant. Figure 8 depicts the shape of $\phi_{k}\left(\beta \right)$ at $k^{*}=1.5$, close to the break-up point $k_{c}≃1.57$.

From the functional form of the eigenfunctions (55) and (56), the dynamical mechanism associated with the spectral break-up becomes clear. As the eigenvalue $\mu^{*}$ approaches −1 from below, the associated eigenfunction develops a singularity at β=0, $\psi_{k}\left(\beta \right)\to 1/\beta$, and consequently the critical point $k_{c}$ corresponds to the bifurcation point where $\psi_{k}\left(\beta \right)$ ceases to be summable.

The occurrence of a single eigenfunction, restricted solely to the interval $\left[-k_{c},k_{c}\right]$, is manifestly incomplete in order to represent a generic (square-summable) function of the internal variable β in the interval [−1,1]. It is therefore interesting to analyze the relaxation properties of the operator $L_{\beta }$, restricted to a planar wave mode $p\left(x,t;\beta \right)=e^{i\;k\;x}\;\zeta \left(\beta ,t\right)$, i.e., to consider the following dynamics:

(57) $$\frac{\partial \zeta (\beta ,t)}{\partial t}=-i\;k^{*}\;\beta \;\zeta \left(\beta ,t\right)-\zeta \left(\beta ,t\right)+\frac{1}{2}\int_{-1}^{1}\zeta \left(\beta^{\prime },t\right)\;d\beta^{\prime }\;,$$

where ζ(β,t) is complex-valued, and the time variable *t* has been made non-dimensional by normalizing it with $\lambda_{0}$. It thus follows from Equation (57) that:

(58) ∂|ζ(β,t)|2∂t=ζ¯(β,t)∂ζ(β,t)∂t+ζ(β,t)∂ζ¯(β,t)∂t=−2|ζ(β,t)|2+Reζ¯(β,t)∫−11ζ(β′,t)dβ′,

where Re[·] is the real part of the argument, and $\bar{\zeta }\left(\beta ,t\right)$ the complex conjugate of ζ(β,t). Integrating over β, the dynamics of the $L^{2}$-norm of ζ(β,t), ${\left||\zeta |\right|}^{2}\left(t\right)=\int_{-1}^{1}{\left|\zeta \left(\beta ,t\right)\right|}^{2}\;d\beta$ is as follows:

(59) $$\frac{{d||\zeta ||}^{2}\left(t\right)}{dt}=-{2\;||\zeta ||}^{2}\left(t\right)+{\left|Z\left(t\right)\right|}^{2}\;,$$

where $Z\left(t\right)=\int_{-1}^{1}\zeta \left(\beta ,t\right)\;d\beta$. As ${\left|Z\left(t\right)\right|}^{2}\geq 0$, it follows from Equation (59) that:

(60) $$\frac{{d||\zeta ||}^{2}\left(t\right)}{dt}\geq -{2\;||\zeta ||}^{2}\left(t\right)\;\Rightarrow \;||\zeta ||\left(t\right)\geq ||\zeta ||\left(0\right)\;e^{-t}\;.$$

Equation (60) indicates that all the planar-wave modes cannot decay to zero faster than $e^{-\gamma^{*}\;t}$, with $\gamma^{*}=1$ representing the maximum relaxation rate. This result is valid for any $k^{*}$, below and above the critical value $k_{c}$.

**Figure 9:**
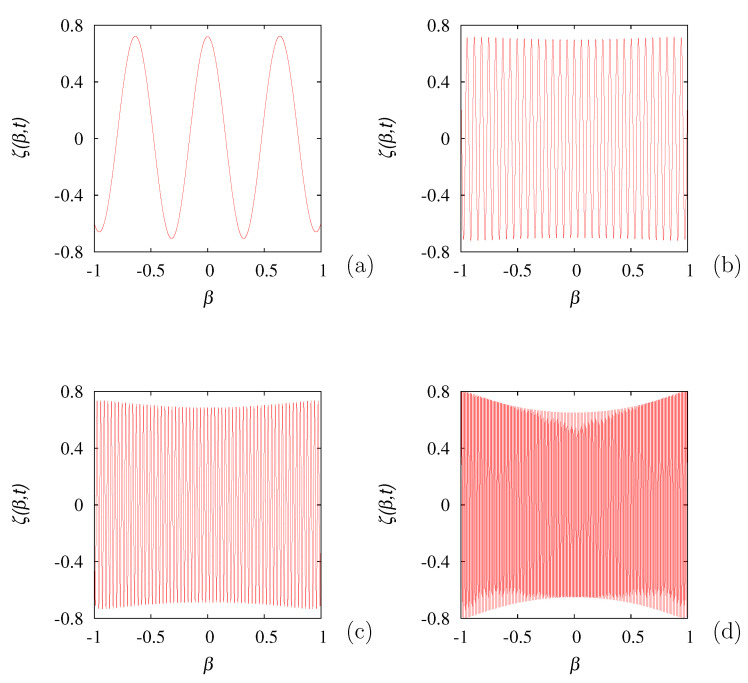
ζ(β,t) vs. β at different time instants for $k^{*}=10$ obtained by solving Equation (57), starting from a uniform initial profile. (**a**) refers to t=1, (**b**) to t=10, (**c**) to t=20, (**d**) to t=50.

Consequently, in the statistical evolution of the process, the point spectrum plays a marginal role, especially for high wave vectors, as the dynamics is controlled by the essential spectral component. From Equation (60), it also follows that the essential spectrum is lower bounded, and this fact is expressed by the property $\gamma^{*}=1$. The typical evolution of a high-wave vector excitation above the critical value $k_{c}$ is depicted in Figure 9, showing the progressive development of a complex structure in β, which does not converge to any proper eigenfunction.

**Figure 10:**
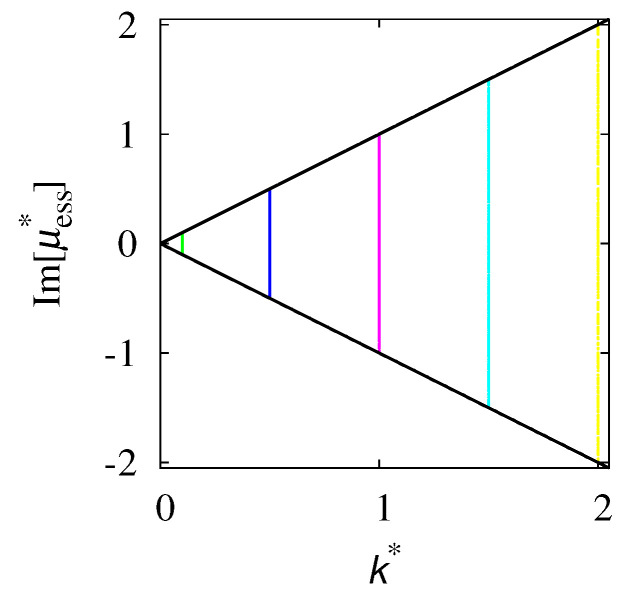
Imaginary part of the essential eigenvalues Im[μess*] vs. $k^{*}$. Horizontal dots correspond to the calculated eigenvalues at $k^{*}=0.01,\;0.1,\;0.5,\;1,\;1.5,\;2$. Solid lines represent the lines Im[μess*]=±k*.

By expanding ζ(β,t) in the truncated Fourier series with respect to β, ζ(β,t)=$\sum_{n=-N}^{N}\zeta_{n}\left(t\right)\;e^{i\;n\;\pi \beta }$, keeping *N* sufficiently large, the eigenvalue spectrum can be calculated by solving the eigenvalue problem $\sum_{k=-N}^{N}M_{n,k}\;\zeta_{k}=\mu^{*}\;\zeta_{n}$, where $M_{0,0}=0$, $M_{n,k}=-k^{*}\;d\left(k-n\right)/2-\delta_{n,k}$, for |n|+|k|>0, where $d\left(k\right)=2{\left(-1\right)}^{k}/\left(k\;\pi \right)$, |k|>0. The direct numerical calculation of the spectrum, obtained numerically by setting N=5000, provides for $k^{*}<k_{c}$, only a single eigenvalue in the point spectrum, as already found. Meanwhile, for all the values of $k^{*}$, the essential spectrum is formed by essential eigenvalues $\mu_{\mathrm{ess}}^{*}$ possessing a real part that is identical and equal to −1, and an imaginary part distributed in an uniform way within the interval $\left[-k^{*},k^{*}\right]$. This phenomenon is depicted in Figure 10.

**Figure 11:**
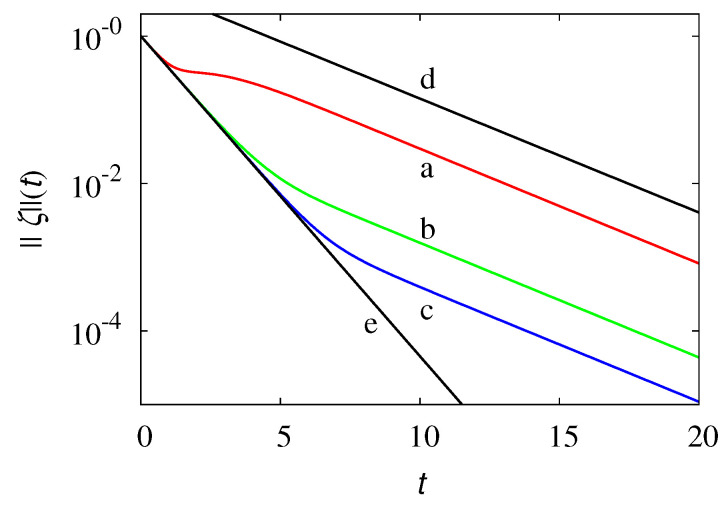
||ζ||(t) vs. *t* at $k^{*}=1$ for several initial conditions, ζ(β,0)=sin(νπβ). Lines (a)–(c) refer to ν=1,10,40, respectively. Line (d) represents the decay $||\zeta ||\left(t\right)∼e^{-r\;t}$ controlled by the point-spectrum eigenvalue r≃0.356, line (e) the decay $||\zeta \left(t\right)||\left(t\right)∼e^{-t}$ associated with the essential spectrum.

The overall dynamics of the process involves both the spatial evolution (i.e., the dynamics with respect to the variable *x*), parametrized by the wave vector *k* (upon a Fourier transform), and the internal dynamics with respect to the variable β, describing the recombination process among the partial density waves. Below the critical threshold $k_{c}$, the unique point spectrum eigenvalue controls the long-term exponential relaxation of the solutions of Equation (57). However, the initial/intermediate decay is sensitive to the relaxation exponent $\gamma^{*}$ that pertains to the essential spectrum. This initial/intermediate behavior becomes more evident, starting from initial conditions ζ(β,0) that possess high-frequency components with respect to the internal variable β. This phenomenon can be clearly observed from the data in Figure 11, which shows the decay of the $L^{2}$-norm ||ζ||(t) of ζ(β,t) at $k^{*}=1$, starting from differential initial sinusoidal profiles, ζ(β,0)=sin(νπβ), ν integer.

For ν∼O(1), only the long-term exponential relaxation can be observed (line a), the exponent of which is the single point-spectrum exponent r≃0.356. As ν increases, $\nu >10^{1}$, a crossover between the initial/intermediate exponential scaling that depends on the essential component of the spectrum $||\zeta ||\left(t\right)∼e^{-\gamma^{*}\;t}$ and on the long-term relaxation $||\zeta ||\left(t\right)∼e^{-rt}$ occurs.

As a final observation, let us consider the decay of the impulsive component of the overall density function for an initial preparation of the system, with particles possessing one and the same initial velocity, i.e., $p\left(x,t;\beta \right)=\delta \left(x\right)\;\delta \left(\beta -\beta^{*}\right)$. This situation corresponds qualitatively to the evolution of the density profiles depicted in Figure 2a for the simpler one-velocity model. In this case, the overall density P(x,t) at time *t* is the superposition of a continuous distribution $P_{c}\left(x,t\right)$ and of an impulsive component centered at $x=b_{0}\;\beta^{*}\;t$:

(61) $$P\left(x,t;\right)=P_{c}\left(x,t\right)+I_{\beta^{*}}\left(t\right)\;\delta \left(x-b_{0}\;\beta^{*}\;t\right)\;.$$

The impulsive component $I_{\beta^{*}}\left(t\right)$, translating in space at velocity $\beta^{*}$, corresponds to the fraction of particles that remain in the initial state $\beta^{*}$, i.e., not experiencing any transition up to time *t*. Because of its physical meaning, it is clear that the relaxation of $I_{\beta^{*}}\left(t\right)$ should not depend on the spatial component of the dynamics of p(x,t;β), i.e., should be independent of the wave vector *k*. Furthermore, it follows from the above observation that this is exclusively a property of the Markov recombination mechanism of the partial density waves, as described by the internal state dynamics of Equation (40), and associated with the internal state operator $\hat{L}$:

(62) $$\hat{L}\left[f\left(\beta \right)\right]=-\lambda_{0}\;f\left(\beta \right)+\frac{\lambda_{0}}{2}\int_{-1}^{1}f\left(\beta^{\prime }\right)\;d\beta^{\prime }\;.$$

The spectrum of $\hat{L}$ is completely degenerate: it possesses only the single eigenvalue $\mu =\lambda_{0}$ with countable multiplicity, as any function π(β) possessing a zero mean in [−1,1] is an eigenfunction associated with it. More specifically, a basis for this eigenspace is given by the sinusoidal functions $\pi_{\nu }\left(\beta \right)=\sin\left(\nu \;\pi \;\beta \right)$, where ν=1,2,… This eigenproperty of the recombination mechanism, when embedded into the spatio-temporal evolution of the partial density waves p(x,t,β) defined by Equation (43), produces results incompatible with the translation operator $-b_{0}\;\beta \;\nabla$ in order to determine any proper eigenstructure, thus contributing to the essential part of the spectrum of the operator $L_{\beta }$ associated with the spatial-temporal evolution of the partial density waves.

**Figure 12:**
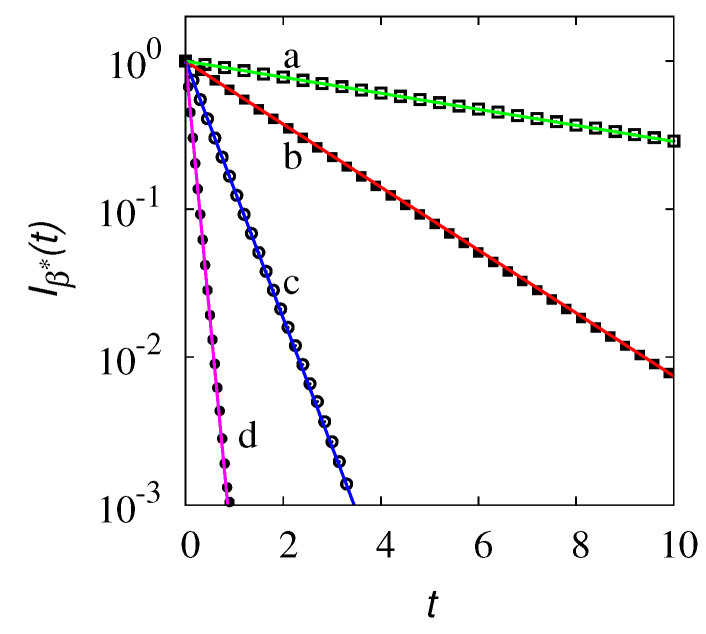
Intensity of the impulsive contribution $I_{\beta^{*}}\left(t\right)$ vs. *t* obtained from stochastic simulations (symbols) at $\beta^{*}=1/2$, for different values of $b_{0}$, keeping constant the ratio $b_{0}^{2}/2\lambda_{0}=1$. The solid lines represent the scaling $I_{\beta^{*}}\left(t\right)=e^{-\lambda_{0}\;t}$. Line (a) and symbols (□) refer to $b_{0}=0.5$, line (b) and symbols (*■*) to $\beta^{*}=1$, line (c) and symbols (∘) to $\beta^{*}=2$, line (d) and symbols (•) to $\beta^{*}=4$.

It follows from the above discussion that the impulsive part of the dynamics $I_{\beta^{*}}\left(t\right)$ entering Equation (62) should decay exponentially as follows:

(63) $$I_{\beta^{*}}\left(t\right)∼e^{-\lambda_{0}\;t}\;.$$

corresponding to the decay defined by the essential spectral component. This phenomenon is depicted in Figure 12. The numerical data have been obtained from the stochastic simulation of an ensemble of $N_{p}=10^{8}$ particles evolving in space according to Equation (43), initially placed at x=0 and all possessing the same velocity $b_{0}\;\beta^{*}$. Therefore, this preparation of the system corresponds to the initial condition for the partial density waves expressed by $p\left(x,0;\beta \right)=\delta \left(x\right)\;\delta \left(\beta -\beta^{*}\right)$. By tracking the evolution of the particle system, the intensity of the impulsive peak $I_{\beta^{*}}\left(t\right)$ can be determined.

Data in Figure 12 refer to different values of $b_{0}$, keeping them fixed at the ratio of $b_{0}^{2}/2\lambda =1$, so that the decay of the impulsive peak depends on the velocity $b_{0}$ as $I_{\beta^{*}}\left(t\right)∼e^{-b_{0}^{2}\;t/2}$.

## 5. Quantum Mechanical Extension

There is an intertwined network of mutual analogies— between the theory of Brownian motion (and more generally stochastic dynamics) and quantum mechanics— that has led to fruitful physical insights and useful mathematical representations, borrowing ideas and techniques from one field to the other [49,50,51,52]. More specifically, there is a strong connection between stochastic processes that possess a finite propagation velocity and quantum mechanics whenever the relativistic constraint on the upper bound for velocities in the space-time propagation of physical phenomena is taken into account. This was already observed by Feynman [53] in his development of the path-integral formulation of quantum mechanics as regards the path of a relativistic free particle (see Figure 2.4 in [53]). However, Gaveau et al. [37] provided the derivation of the strict analogy between the 1+1 Dirac equation (1 spatial dimension + 1 temporal dimension) and the Poisson–Kac processes in the presence of imaginary transition rates, i.e.,:

(64) $$\begin{array}{ccc} \frac{\partial \psi_{+}\left(x,t\right)}{\partial t} & = & -c\;\frac{\partial \psi_{+}\left(x,t\right)}{\partial t}+i\;\lambda_{0}\;\left[\psi_{+}\left(x,t\right)-\psi_{-}\left(x,t\right)\right]\\ \frac{\partial \psi_{-}\left(x,t\right)}{\partial t} & = & c\;\frac{\partial \psi_{-}\left(x,t\right)}{\partial t}+i\;\lambda_{0}\;\left[\psi_{-}\left(x,t\right)-\psi_{+}\left(x,t\right)\right]\;, \end{array}$$

where *c* is the velocity of light *in vacuo*, and $i=\sqrt{-1}$ and $\left(\psi_{+},\psi_{-}\right)$ are the two components of the vector-valued wave function. The spatial probability density is $|\psi_{+}{|}^{2}+{\left|\psi_{-}\right|}^{2}$, consistent with the Dirac theory [54]. As for the probabilistic theory of Poisson–Kac processes, Equation (64) converges towards the Schrödinger equation in the limit of $c,\lambda_{0}\to \infty$, keeping fixed the ratio $c^{2}/2\lambda_{0}$ (Kac limit). This model has been analyzed and generalized by several authors [55,56,57,58,59].

In this Section, we extend the stochastic model considered in the previous paragraphs to a quantum mechanical setting through the approach used by Gaveau et al. [37] discussed above, by considering a continuous velocity instead of a single velocity *c*, as in (64). The reason for this extension stems from the observation that the complete description of the dynamics of the stochastic process (43) requires the introduction of the addition variable β parametrizing the partial density functions p(x,t;β). In a quantum extension, this variable can be viewed as a “hidden variable” associated with a sub-quantum level of description of the system, from which classical quantum theory can be viewed as an emergent property. In other words, this quantum extension is aimed at providing an archetype for possible sub-quantum descriptions of reality, in the spirit of the work by David Bohm [38,39], which shows how classical quantum theory can be emergently derived from this “hidden” level. As we are primarily interested in highlighting these two aspects, the simple case of a free particle is considered in a one-dimensional spatial setting, in agreement with the stochastic model developed in the previous Sections.

The quantum mechanical counterpart of Equation (43) is given by the following equation:

(65) $$\frac{\partial \psi (x,t;\beta )}{\partial t}=-b_{0}\;\beta \;\frac{\partial \psi (x,t;\beta )}{\partial x}+i\;\lambda_{0}\;\left[\psi \left(x,t;\beta \right)-\frac{1}{2}\int_{-1}^{1}\psi \left(x,t;\beta^{\prime }\right)\;d\beta^{\prime }\right]\;,$$

where ψ(x,t;β) is the wavefunction at the subquantum level, parametrized with respect to the additional variable β∈[−1,1]. The constants $b_{0}$ and $\lambda_{0}$ are determined below.

Let Ψ(x,t) be the classical (Schrödinger) wavefunction:

(66) $$\Psi \left(x,t\right)=\int_{-1}^{1}\psi \left(x,t;\beta \right)\;d\beta \;,$$

then introduce the flux $J_{\psi }\left(x,t\right)=b_{0}\;\int_{-1}^{1}\beta \;\psi \left(x,t;\beta \right)\;d\beta$, such that:

(67) $$\frac{\partial \Psi (x,t)}{\partial t}=-\frac{\partial J_{\psi }\left(x,t\right)}{\partial x}\;.$$

From Equation (65), it follows that $J_{\psi }\left(x,t\right)$ fulfills the following equation: Equation

(68) $$\frac{\partial J_{\psi }\left(x,t\right)}{\partial t}=-b_{0}^{2}\;\frac{\partial }{\partial x}\int_{-1}^{1}\beta^{2}\;\psi \left(x,t;\beta \right)\;d\beta +i\;\lambda_{0}\;J_{\psi }\left(x,t\right)\;.$$

Let us consider the Kac limit of this model, letting $b_{0},\;\lambda_{0}\to \infty$ and keeping constant the nominal diffusivity $b_{0}^{2}/2\lambda_{0}=D_{\mathrm{nom}}$. From Equation (65), in the Kac limit, we have the following:

(69) $$\psi \left(x,t;\beta \right)=\frac{1}{2}\int_{-1}^{1}\psi \left(x,t;\beta \right)\;d\beta =\frac{\Psi (x,t)}{2}\;,$$

i.e., ψ(x,t;β) in the Kac limit does not depend on β, while $J_{\psi }\left(x,t\right)$ attains the following expression:

(70) $$J_{\psi }\left(x,t\right)=-i\frac{b_{0}^{2}}{2\;\lambda_{0}}\;2\frac{\partial }{\partial x}\int_{-1}^{1}\beta^{2}\;\psi \left(x,t;\beta \right)\;d\beta =-i\frac{2\;D_{\mathrm{nom}}}{3}\;\frac{\partial \Psi (x,t)}{\partial x}\;,$$

where Equation (69) has been used. From Equation (70) inserted in Equation (67), it follows that if $D_{\mathrm{nom}}$ is such that $D_{\mathrm{nom}}=3\;D_{\hbar }/2$, where $D_{\hbar }=\hbar /2\;m$ is the quantum diffusivity for a massive particle of mass *m*, then Ψ(x,t) satisfies the Schrödinger equation:

(71) $$-i\;\frac{\partial \Psi (x,t)}{\partial t}=D_{\hbar }\;\frac{\partial^{2}\psi \left(x,t\right)}{\partial x^{2}}\;.$$

Moreover, it is easy to show that Equation (65) is a proper quantum mechanical equation, in the sense that a Born rule can be defined from it. In fact, elementary calculations provide the following:

(72) $$\frac{\partial }{\partial t}\int_{-1}^{1}{\left|\psi \left(x,t;\beta \right)\right|}^{2}\;d\beta =-\frac{\partial }{\partial x}\left(b_{0}\;\int_{-1}^{1}\beta {\left|\psi \left(x,t;\beta \right)\right|}^{2}\;d\beta \right)\;.$$

Therefore, $\int_{-1}^{1}{\left|\psi \left(x,t;\beta \right)\right|}^{2}\;d\beta$ is a conserved quantity corresponding to the quantum probability density function with respect to the position *x*. Observe that in the Kac limit, ψ(x,t;β)∼Ψ(x,t), and therefore $\int_{-1}^{1}{\left|\psi \left(x,t;\beta \right)\right|}^{2}\;d\beta ∼{\left|\Psi \left(x,t\right)\right|}^{2}$, which is in agreement with Schrödinger’s non-relativistic quantum theory.

For the sake of completeness, it is useful to observe that Equation (65) refers to a single free particle, but its extension to include the action of a potential or its generalization to a many-body problem is straightforward. If U(x) is a potential acting on a massive particle, the associated quantum model generalizing Equation (65) is expressed by the following equation:

(73) $$\begin{array}{ccc} \frac{\partial \psi (x,t;\beta )}{\partial t} & = & -b_{0}\;\beta \;\frac{\partial \psi (x,t;\beta )}{\partial x}-i\;\frac{U(x)}{\hbar }\;\psi \left(x,t;\beta \right)\\ & + & i\;\lambda_{0}\;\left[\psi \left(x,t;\beta \right)-\frac{1}{2}\int_{-1}^{1}\psi \left(x,t;\beta^{\prime }\right)\;d\beta^{\prime }\right]\;. \end{array}$$

Using the same approach outlined above, it is easy to see that Equation (65) converges in the Kac limit of the non-relativistic Schrödinger equation in the presence of the potential U(x). In a similar way, the extension to a many-body problem is formally simple. To begin with, consider a single spatial dimension. Let $x=(x_{1},\ldots ,x_{n})$ be the coordinate vector of the particle system, where $x_{k}$ is the position of the *k*-th particle, k=1,⋯,n, and $\beta =(\beta_{1},\cdots ,\beta_{n})$, $\lambda =(\lambda_{1},\cdots ,\lambda_{n})$, so that $b_{0}\;\beta_{k}$ is the velocity of the *k*-th particle with $\beta_{k}\in \left[-1,1\right]$, and $\lambda_{k}$ is its transition rate. The extension of Equation (65) to an *n*-body problem on the real line in the presence of the interaction potential U(x) reads as follows:

(74) $$\begin{array}{ccc} \frac{\partial \psi (x,t;\beta )}{\partial t} & = & -b_{0}\;\sum_{k=1}^{n}\beta_{k}\;\frac{\partial \psi (x,t;\beta )}{\partial x_{k}}-i\;\frac{U(x)}{\hbar }\psi \left(x,t;\beta \right)\\ & + & i\;\sum_{k=1}^{n}\lambda_{k}\;\left[\psi \left(x,t;\beta \right)-\frac{1}{2^{n}}\int_{-1}^{1}\cdots \int_{-1}^{1}\psi \left(x,t;\beta^{\prime }\right)\;d\beta^{\prime }\right]\;, \end{array}$$

where $d\beta^{\prime }=d\beta_{1}^{\prime }\cdot \cdots \cdot d\beta_{n}^{\prime }$. In higher dimensions, considering only translational motion, let us define $X=(x_{1},\cdots ,x_{n})$, where $x_{k}=\left(x_{k,1},\cdots ,x_{k,d}\right)$ is the position vector of the *k*-th particle in a *d*-dimensional space, and $\bar{\beta }=\left(\beta_{1},\cdots ,\beta_{n}\right)$, where the velocity of the *k*-th particle is $b_{0}\;\beta_{k}=b_{0}\left(\beta_{k,1},\cdots ,\beta_{k,d}\right)$. Thus, assuming isotropic transition rates, Equation (74) simply becomes:

(75) $$\begin{array}{ccc} \frac{\partial \psi (X,t;\bar{\beta })}{\partial t} & = & -b_{0}\;\sum_{k=1}^{n}\beta_{k}\cdot \nabla_{k}\psi \left(X,t;\bar{\beta }\right)-i\;\frac{U(X)}{\hbar }\;\psi \left(X,t;\bar{\beta }\right)\\ & + & i\;\sum_{k=1}^{n}d\;\lambda_{k}\;\left[\psi \left(X,t;\bar{\beta }\right)-\frac{1}{2^{n\;d}}\int_{-1}^{1}\cdots \int_{-1}^{1}\psi \left(X,t;{\bar{\beta }}^{\prime }\right)\;d{\bar{\beta }}^{\prime }\right]\;, \end{array}$$

where $\nabla_{k}=\left(\partial /\partial x_{k,1}\cdots \partial /\partial x_{k,d}\right)$ and $d{\bar{\beta }}^{\prime }=\prod_{k=1}^{n}\prod_{h=1}^{d}d\beta_{k,h}^{\prime }$. The inclusion of *n*-body interactions expressed by the potential U(X) is perfectly admissible in a non-relativistic framework, while it would require care and attention from a relativistic perspective. But this discussion is manifestly outside the scope of the present work.

### Spectral Properties and Dispersion Relations

Let us consider the spectral properties of the generalized Schrödinger operator,

(76) $$L_{q}\left[\phi \left(x,\beta \right)\right]=-b_{0}\;\beta \frac{\partial \psi (x,\beta )}{\partial x}+i\;\lambda_{0}\left[\psi \left(x,\beta \right)-\frac{1}{2}\int_{-1}^{1}\psi \left(x,\beta^{\prime }\right)\;d\beta^{\prime }\right]\;,$$

and let ε be an eigenvalue, $L_{q}\left[\phi \left(x,\beta \right)\right]=\varepsilon \;\phi \left(x,\beta \right)$. The eigenfunctions are of the form $\phi \left(x,\beta \right)=e^{i\;k\;x}\;\psi_{0}\left(\beta \right)$. As before, by introducing the normalized quantities $\varepsilon^{*}=\varepsilon /\lambda_{0}$, $k^{*}=k\;b_{0}/\lambda_{0}$, it thus follows that that the “internal component” of the eigenfunction $\phi_{0}\left(\beta \right)$ associated with the normalized eigenvalue $\varepsilon^{*}$ is given by the following equation:

(77) $$\phi_{0}\left(\beta \right)=\frac{A}{\varepsilon^{*}-1+k^{*}\;\beta }\;,$$

where *A* is a constant, leading to the following eigenvalue equation:

(78) $$-2=\int_{-1}^{1}\frac{d\beta }{\varepsilon^{*}-1+k^{*}\;\beta }\;,$$

which provides, upon quadraturae and algebraic manipulations, the expression for the eigenvalue:

(79) $$\varepsilon^{*}=1-\frac{k^{*}\;\left(1+e^{-2\;k^{*}}\right)}{1-e^{-2\;k^{*}}}\;.$$

In the quantum case, for any wave vector *k*, there exists an eigenvalue in the point spectrum of the operator $L_{q}$, which is not the case for its probabilistic counterpart $L_{\beta }$, as thoroughly analyzed in Section 4.1.

Let us consider the scaling properties of the eigenvalue spectrum, i.e., the dispersion relation connecting the particle energy E=−ℏε to the wave vector *k*. The Kac limit ($b_{0},\;\lambda_{0}\to \infty$, $b_{0}^{2}/2\;\lambda_{0}=\mathrm{constant}$) corresponds to small values of $k^{*}$. Expanding the exponential displayed in Equation (79) in a Taylor series up to the leading order, in this case $e^{-2\;x}=1-2x+2x^{2}-4x^{3}/3$, and by developing the necessary algebra, one obtains the following from Equation (79):

(80) $$\varepsilon^{*}=-\frac{{\left(k^{*}\right)}^{2}}{3}+O\left({\left(k^{*}\right)}^{3}\right)\;.$$

Neglecting the $O({\left(k^{*}\right)}^{3})$ term, it implies the following for the eigenvalue ε:

(81) $$\varepsilon =\lambda_{0}\;\varepsilon^{*}=-\frac{\lambda_{0}}{3}\;\frac{b_{0}^{2}\;k^{2}}{\lambda_{0}^{2}}=-\frac{2\;D_{\mathrm{nom}}}{3}\;k^{2}=-D_{\hbar }\;k^{2}\;,$$

consequently giving the energy as follows:

(82) $$E=-\hbar \;D_{\hbar }\;k^{2}=\frac{\hbar^{2}\;k^{2}}{2\;m}\;,$$

which is consistent with the classical quantum mechanical result.

Consider the quantity $-\varepsilon /D_{\hbar }$, which in the classical quantum limit is equal to $k^{2}$, and set $\sigma =\lambda_{0}/D_{\hbar }$. Since $k^{*}=\sqrt{3/\sigma }\;k$ it follows that:

(83) $$-\frac{\varepsilon }{D_{\hbar }}=-\sigma \;{\left[\frac{\left(1-e^{2\;k^{*}}\right)-k^{*}\;\left(1+e^{-2\;k^{*}}\right)}{1-e^{-2\;k^{*}}}\right]}_{k^{*}=\sqrt{3/\sigma }\;k}\;.$$

For large values, |k|, $-\varepsilon /D_{\hbar }=\sigma \left|k^{*}\right|$, and therefore $-\varepsilon /D_{\hbar }$ admits the following crossover behavior:

(84) $$-\frac{\varepsilon }{D_{\hbar }}=\left\{\begin{array}{ccc} k^{2}\;, & \; & |k|<\sqrt{3\;\sigma }\\ \sqrt{3\;\sigma }\;\left|k\right|\;, & \; & |k|>\sqrt{3\;\sigma } \end{array}\right.\;.$$

**Figure 13:**
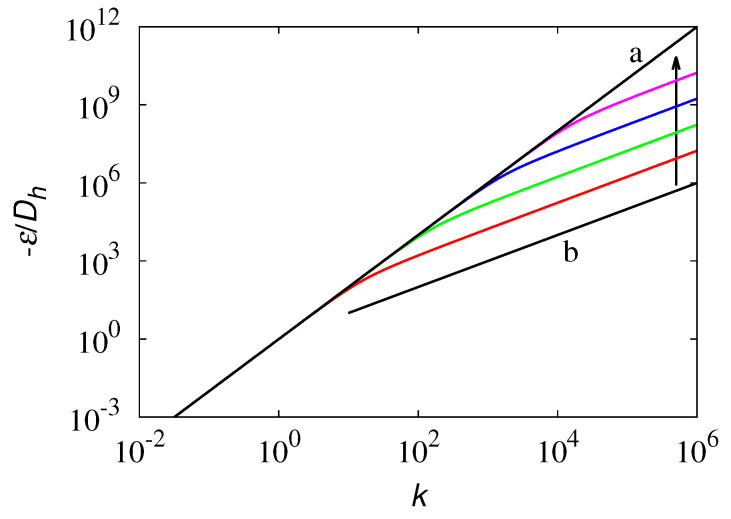
Dispersion curve $-\varepsilon /D_{h}$ vs. the wavenumber |k| for a free particle in the sub-quantum model (65). Solid line (a) is the classical quantum result $-\varepsilon /D_{h}=k^{2}$. Solid line (b) represents the linear scaling $-\varepsilon /D_{h}∼\left|k\right|$. The other lines depict the graph of the function at the r.h.s. of Equation (83) for increasing values of $\sigma =10^{2},\;10^{4},\;10^{6},\;10^{8}$, indicated by the direction of the arrow.

This phenomenon is depicted in Figure 13. The linear scaling with |k| in the high wave vector region is the signature in the quantum model of the bounded propagation velocity characterizing the generalized Schrödinger operator $L_{q}$ for finite values of $b_{0}$ and $\lambda_{0}$.

## 6. Concluding Remarks

In this article, we have analyzed the spectral (eigenvalue) properties of several classes of stochastic processes that possess a finite propagation velocity, showing the occurrence of a lower bound for the real part of the eigenvalue spectrum. As regards this spectral property, it is immaterial whether the long-term diffusive behavior is regular (linear Einsteinian scaling of the mean square displacement) or anomalous (superdiffusive). This result marks a fundamental and significant difference with respect to the parabolic diffusion model, for which short wavelength perturbations decay at a rate proportional to the squared norm of the wave vector.

This result has several general implications. (i) In the approach to the foundations of the thermodynamics of irreversible processes [60]: The results obtained confirm the analysis developed in [61], indicating that the Markov operators associated with the SEOs of stochastic processes characterized by a finite propagation velocity are invertible and form a group of transformations parametrized with respect to time *t*. Conversely, in the case of parabolic diffusion models, the weaker semi-group property for t≥0 holds. (ii) In classical field and transport theory: The results indicate that the hyperbolic field equations (e.g., for mass, heat, and momentum transfer), which can be developed starting from microscopic equations of motions expressed in the form of stochastic equations and characterized by a bounded velocity of propagation [47], may have completely different stability properties than their parabolic counterparts.

The occurrence of a lower bound for the real part of the eigenspectrum of operators describing the statistical evolution of stochastic processes possessing a finite propagation velocity also occurs for continuum models with a spectrum of particle velocities. In this article, we considered a prototypical example on the real line, in which the relaxational properties at high wave vectors are entirely associated with the essential part of the spectrum. The lower spectral bound is a consequence of the internal recombination dynamics of the stochastic states of the system and corresponds to the largest decay exponent (with reversed sign) of the overall probability density function P(x,t), for an initial configuration with all the particles located at the same point and possessing the same velocity.

From a broader perspective, the analysis of SEOs outlined in this article can be extended to other relevant physical phenomenologies. This is the case for the linear response of stochastic systems that possess anomalous behavior, addressed in [62,63,64,65,66] by either using Continuous Time Random Walk scalings or approximate SEOs involving fractional derivative operators. The same problem can be framed within the age formalism of LWs introduced in Section 3, by keeping the same definitions and evolution in Equations (7)–(10), and modifying the boundary condition of Equation (11) in the following form:

(85) $$p_{\pm }\left(x,t;0\right)=A_{\pm ,+}\left(t\right)\;\int_{0}^{\infty }\lambda \left(\tau \right)\;p_{+}\left(x,t;\tau \right)\;d\tau +A_{\pm ,-}\left(t\right)\;\int_{0}^{\infty }\lambda \left(\tau \right)\;p_{-}\left(x,t;\tau \right)\;d\tau \;,$$

where the transition probabilities $A_{\pm ,\pm }\left(t\right)$, $A_{\pm ,\pm }\left(t\right)\geq 0$, $A_{+,\pm }\left(t\right)+A_{-,\pm }\left(t\right)=1$ account for the effect of the forcing field f(t) and are defined according to [62,65] as follows:

(86) $$A_{+,\pm }\left(t\right)=\frac{1+\mu \;f(t)}{2}\;,\;A_{-,\pm }\left(t\right)=\frac{1-\mu \;f(t)}{2}\;,$$

where 0<μ<1 and |f(t)|≤1. In the analysis of this problem, the spectral results outlined in this manuscript are of limited use, as the simplest way to tackle this problem from physical grounds is to consider the partial moment hierarchy $m_{\pm }^{\left(n\right)}=\int_{-\infty }^{\infty }x^{n}\;p_{\pm }\left(x,t;\tau \right)\;dx$, which will be addressed in forthcoming works.

Getting back to our probabilistic model with a spectrum of velocities, its quantum extension displays two main qualitative properties: (i) Contrary to the purely probabilistic case, for any k∈R, an eigenvalue of the quantum operator $L_{q}$ exists; (ii) The boundedness in the propagation velocity characterizing $L_{q}$ implies, in the quantum case, a linear dispersive relation of the energy *E* with respect to |k| for high values of *k*. This result is a direct consequence of the undulatory propagation, as from quantization E=ℏω, and the ω is related to the group velocity $v_{g}$, by $d\omega /dk=v_{g}$, implying E∼k. The quantum model analyzed in Section 4.1 is a simple example of a sub-quantum model possessing internal (“hidden”) degrees of freedom (in this case, the internal variable β∈[−1,1]) and providing the same emergent results as classical quantum theory.

## Data Availability

Not applicable.

## Abbreviations

The following abbreviations are used in this manuscript:

SEO	Statistical evolution operator
GPK	Generalized Poisson–Kac
LW	Lévy Walk

## References

[1] Klages R., Just W., Jarzynski C. (2013). Nonequilibrium Statistical Physics of Small Systems: Fluctuation Relations and Beyond.

[2] Bechinger C., di Leonardo R., Löwen H., Reichhardt C., Volpe G., Volpe G. (2016). Active particles in complex and crowded environments. Rev. Mod. Phys..

[3] Marchetti M.C., Joanny J.F., Ramaswamy S., Liverpool T.B., Prost J., Rao M., Simha R.A. (2013). Hydrodynamics of soft active matter. Rev. Mod. Phys..

[4] Klages R., Radons G., Sokolov I.M. (2008). Anomalous Transport: Foundations and Applications.

[5] Krapivsky P.L., Redner S., Ben-Naim E. (2010). A Kinetic View of Statistical Physics.

[6] Ebeling W., Sokolov I.M. (2005). Statistical Thermodynamics and Stochastic Theory of Nonequilibrium Systems.

[7] Viswanathan G.M., da Luz M.G.E., Raposo E.P., Stanley H.E. (2011). The Physics of Foraging.

[8] Müller I. (1999). Speeds of propagation in classical and relativistic extended thermodynamics. Living Rev. Relativ..

[9] Giona M. (2017). Relativistic analysis of stochastic kinematics. Phys. Rev. E.

[10] Wolschin G. (2007). Diffusion in relativistic systems. Prog. Particle Nucl. Phys..

[11] Muniz C.R., Cunha M.S., Filho R.N.C., Berezza V.B. (2015). Some remarks on relativistic diffusion and the spectral dimension criterion. Phys. Rev. D.

[12] Müller I., Ruggeri T. (1993). Extended Thermodynamics.

[13] Ferreira J.A., Grassi M., Gudino E., de Oliveira P. (2015). A new look to non-Fickian diffusion. Appl. Math. Model.

[14] Jou D., Cimmelli V.A. (2016). Constitutive equations for heat conduction in nanosystems and nonequilibrium processes: An overview. Comm. Appl. Ind. Math..

[15] Kac M. (1974). A stochastic model related to the telegrapher’s equation. Rocky Mount. J. Math..

[16] Pinsky M.A. (1991). Lectures on Random Evolutions.

[17] Kolesnik A.D. (2007). A note on planar random motion at finite speed. J. Appl. Prob..

[18] Giona M., Crescitelli S., Brasiello A. (2017). Stochastic foundations of undulatory transport phenomena: Generalized Poisson–Kac processes—Part I basic theory. J. Phys. A.

[19] Shlesinger M.F., Klafter J., Wong Y.M. (1982). Random walks with infinite spatial and temporal moments. J. Stat. Phys..

[20] Shlesinger M.F., Klafter J., West B.J. (1986). Lévy walks with applications to turbulence and chaos. Physica A.

[21] Klafter J., Blumen A., Shlesinger M.F. (1987). Stochastic pathway to anomalous diffusion. Phys. Rev. A.

[22] Zaburdaev V., Denisov S., Klafter J. (2015). Lévy walks. Rev. Mod. Phys..

[23] Jou D., Casas-Vazquez J., Lebon G. (2001). Extended Irreversible Thermodynamics.

[24] Hänggi P., Jung P. (1995). Colored noise in dynamical systems. Adv. Chem. Phys..

[25] Weiss G.H. (1994). Aspects and Applications of the Random Walk.

[26] Horsthemke W., Lefever R. (1984). Noise-Induced Transitions.

[27] Bena I. (2006). Dichotomous Markov noise: Exact results for out-of-equilibrium systems. Int. J. Mod, Phys. B.

[28] d’Onofrio A. (2013). Bounded Noises in Physics, Biology and Engineering.

[29] Fedotov S. (2016). Single integrodifferential wave equation for a Lévy walk. Phys. Rev. E.

[30] Fedotov S., Korabel N. (2017). Emergence of Lévy walks in systems of interacting individuals. Phys. Rev. E.

[31] Stage H. (2017). Aging in mortal superdiffusive Lévy walkers. Phys. Rev. E.

[32] Shkilev V.P. (2018). Subordinated stochastic processes with aged operational time. Phys. Rev. E.

[33] Giona M., D’Ovidio M., Cocco M., Cairoli A., Klages R. (2019). Age representation of Lévy walks: Partial density waves, relaxation and first passage time statistics. J. Phys. A.

[34] Giona M., Cairoli A., Klages R. (2020). Extended Poisson-Kac theory: A unifying framework for stochastic processes with finite propagation velocity. arXiv.

[35] Oxenius J. (1986). Kinetic Theory of Particles and Photons.

[36] Kato T. (1995). Perturbation Theory for Linear Operators.

[37] Gaveau B., Jacobson T., Kac M., Schulman L.S. (1984). Relativistic Extension of the Analogy between Quantum Mechanics and Brownian Motion. Phys. Rev. Lett..

[38] Bohm D., Hiley B.J. (1993). The Undivided Universe.

[39] Bohm D. (1980). Wholeness and the Implicate Order.

[40] Kolesnik A.D., Ratanov N. (2013). Telegraph Processes and Option Pricing.

[41] Kolesnik A.D. (2021). Markov Random Flights.

[42] Giona M., Pucci L. (2019). Hyperbolic heat/mass transport and stochastic modelling—Three simple problems. Math. Eng..

[43] Politi M., Kaizoj T., Scalas E. (2011). Full characterization of the fractional Poisson process. Europhys. Lett..

[44] Sheng H., Chen Y., Qiu T. (2011). Fractional Processes and Fractional-Order Signal Processing: Techniques and Applications.

[45] Abramowitz M., Stegun I.A. (2014). Handbook of Mathematical Functions.

[46] Zaburdaev V., Fouxon I., Denisov S., Barkai E. (2016). Superdiffusive Dispersals Impart the Geometry of Underlying Random Walks. Phys. Rev. Lett..

[47] Giona M., Brasiello A., Crescitelli S. (2017). Stochastic foundations of undulatory transport phenomena: Generalized Poisson–Kac processes—Part III extensions and applications to kinetic theory and transport. J. Phys. A.

[48] Giona M., Brasiello A., Crescitelli S. (2017). Stochastic foundations of undulatory transport phenomena: Generalized Poisson–Kac processes—Part II Irreversibility, norms and entropies. J. Phys. A.

[49] Nelson E. (1966). Derivation of the Schrödinger equation from Newtonian mechanics. Phys. Rev..

[50] Nelson E. (1985). Quantum Fluctuations.

[51] Gudder S.P. (2005). Stochastic Methods in Quantum Mechanics.

[52] Nagasawa M. (1993). Schrödinger Equations and Diffusion Theory.

[53] Feynman R.P., Hibbs A.R. (1965). Quantum Mechanics and Path Inregrals.

[54] Thaller B. (1992). The Dirac Equation.

[55] de Angelis G.F., Jona-Lasinio G., Serva M., Zanghi N. (1986). Stochastic mechanics of a Dirac particle in two spacetime dimensions. J. Phys. A.

[56] McKeon D.G.C., Ord G.N. (1992). Time Reversal in Stochastic Processes and the Dirac Equation. Phys. Rev. Lett..

[57] Bialynicki-Birula I. (1994). Weyl, Dirac, and Maxwell equations on a lattice as unitary cellular automata. Phys. Rev. D.

[58] Strauch F.W. (2006). Relativistic quantum walks. Phys. Rev. A.

[59] Balakrishnan V., Lakshmibala S. (2005). On the connection between biased dichotomous diffusion and the one-dimensional Dirac equation. New J. Phys..

[60] Mackey M.C. (2003). Time’s Arrow—The Origins of Thermodynamic Behavior.

[61] Giona M. (2017). Variational principles and Lagrangian functions for stochastic processes and their dissipative statistical descriptions. Physica A.

[62] Sokolov I.M., Blumen A., Klafter J. (2001). Linear response in complex systems: CTRW and the fractional Fokker–Planck equations. Physica A.

[63] Allegrini P., Aquino G., Grigolini P., Palatella L., Rosa A., West B.J. (2005). Correlation function and generalized master equation of arbitrary age. Phys. Rev. E.

[64] Sokolov I.M., Klafter J. (2006). Field-Induced Dispersion in Subdiffusion. Phys. Rev. Lett..

[65] Aquino G., Grigolini P., West B.J. (2007). Linear response and Fluctuation-Dissipation Theorem for non-Poissonian renewal processes. Europhys. Lett..

[66] Aquino G., Bologna M., Grigolini P., West B.J. (2010). Beyond the Death of Linear Response: 1/f Optimal Information Transport. Phys. Rev. Lett..

